# Immune checkpoint blockade induces gut microbiota translocation that augments extraintestinal anti-tumor immunity

**DOI:** 10.1126/sciimmunol.abo2003

**Published:** 2023-03-03

**Authors:** Yongbin Choi, Jake N. Lichterman, Laura A. Coughlin, Nicole Poulides, Wenling Li, Priscilla Del Valle, Suzette N. Palmer, Shuheng Gan, Jiwoong Kim, Xiaowei Zhan, Yajing Gao, Bret M. Evers, Lora V. Hooper, Chandrashekhar Pasare, Andrew Y. Koh

**Affiliations:** 1 Department of Pediatrics, Division of Hematology/Oncology, The University of Texas Southwestern Medical Center, Dallas, TX 75390; 2 Department of Immunology, The University of Texas Southwestern Medical Center, Dallas, TX 75390; 3 Division of Hematology/Oncology, Department of Internal Medicine, The University of Texas Southwestern Medical Center, Dallas, TX 75390, USA.; 4 Department of Cell and Molecular Biology, The University of Texas Southwestern Medical Center, Dallas, TX. 75390; 5 Department of Population and Data Sciences, The University of Texas Southwestern Medical Center, Dallas, TX 75390; 6 Department of Biomedical Engineering, The University of Texas Southwestern Medical, Dallas, TX 75390; 7 Department of Pathology, The University of Texas Southwestern Medical, Dallas, TX 75390; 8 The Howard Hughes Medical Institute, The University of Texas Southwestern Medical Center, Dallas, TX 75390; 9 Division of Immunobiology and Center for Inflammation and Tolerance, Cincinnati Children’s Hospital Medical Center Cincinnati, OH 45229; 10 Department of Pediatrics, University of Cincinnati, College of Medicine, Cincinnati, OH 45220; 11 Harold C. Simmons Comprehensive Cancer Center, The University of Texas Southwestern Medical Center, Dallas, TX 75390; 12 Department of Microbiology, The University of Texas Southwestern Medical Center, Dallas, TX 75390

## Abstract

Gut microbiota, specifically gut bacteria, are critical for effective immune checkpoint blockade therapy (ICT) for cancer. The mechanisms by which gut microbiota augment extraintestinal anti-cancer immune responses, however, are largely unknown. Here, we find that ICT induces the translocation of specific endogenous gut bacteria into secondary lymphoid organs and subcutaneous melanoma tumors. Mechanistically, ICT induces lymph node remodeling and dendritic cell (DC) activation, which facilitates the translocation of a selective subset of gut bacteria to extraintestinal tissues to promote optimal anti-tumor T cell responses in both the tumor-draining lymph nodes (TDLN) and the primary tumor. Antibiotic treatment results in decreased gut microbiota translocation into MLN and TDLN, diminished DC and effector CD8+ T cell responses, and attenuated response to ICT. Our findings illuminate a key mechanism by which gut microbiota promote extraintestinal anti-cancer immunity.

## One sentence summary

Immune checkpoint blockade therapy induces gut bacteria translocation into secondary lymphoid organs and promotes extraintestinal anti-tumor immunity

## Gut Microbiota and Immune Checkpoint Blockade

Recent studies have suggested that the gut microbiome influences responses to immune checkpoint therapy (ICT) for cancer, but the mechanisms driving these responses are not clear. Choi et al. analyzed the translocation of specific endogenous bacteria from the gut to secondary lymphoid organs or melanoma tumors in a murine model. They observed translocation of some bacterial species during ICT enhanced dendritic cell activation and lymph node remodeling. These enhanced responses promoted anti-tumor T cell responses in tumor draining lymph nodes and subcutaneous tumors. In contrast, antibiotic treatment diminished gut microbe translocation to draining lymph nodes and attenuated anti-tumor responses mediated by ICT. These findings provide critical insight into the role of gut microbe translocation in enhancing effects of ICT.

## INTRODUCTION

Immune checkpoint inhibitor therapy (ICT), targeting cytotoxic T-lymphocyte–associated-antigen-4 (CTLA-4) and/or programmed cell death protein 1 (PD-1), results in durable remissions in patients with previously incurable cancers (1). Yet up to 50% of cancer patients remain unresponsive to ICT (2). A variety of host-associated factors have been implicated as a potential cause of this therapeutic discrepancy, and one of the most intriguing is the gut microbiome. Growing evidence suggests that the gut microbiome plays an instructive role in modulating cancer immunotherapy (3–14). Mice that lack gut microbiota or those pre-treated with antibiotics have a dramatically diminished response to ICT (9, 13, 14). Our group (7) and others (4, 8, 12) have identified specific, yet distinct, gut bacteria associated with clinical response to cancer immunotherapy. Recent reports also suggest that fecal microbiota transplantation is both safe and potentially effective in augmenting anti-cancer immune responses in cancer patients previously unresponsive to immunotherapy (15, 16).

There is no consensus on which gut microbiota are required for optimal host anti-cancer immune responses. A variety of gut bacteria species have been shown to correlate with positive clinical responses to immunotherapy and/or can augment ICT responses in preclinical cancer models: *Bifidobacterium* spp. (10, 13), *Akkermansia muciniphila* (12), *Enterococcus* spp. (5, 17), and *Faecalibacterium prausnitzii* (4, 7, 8). To further confound matters, *Bacteroides* species have been shown to have both potentially beneficial (7, 14) and negative effects (16, 18) on cancer immunotherapy responses. Thus, it is unclear as to what generalizable “rules” can be ascertained from these data.

Gut bacteria can augment cancer immunotherapy responses through a variety of mechanisms. Bacteria have long been known to be potent activators of the innate immune system, which can then prime T cells and induce anti-cancer immune responses (e.g. Coley’s Toxin) (19). Gut bacteria-derived metabolites (e.g. inosine (9), c-di-AMP (20) or short-chain fatty acids (3, 21)) or pathogen-associated molecular patterns (e.g. muramyl peptide (22)) can enhance anti-cancer T cell responses. Finally, in some instances, specific endogenous gut bacteria may harbor antigens that cross-react with tumor antigens/neoantigens and may increase T cell mediated anti-cancer immune responses via molecular mimicry (23).

Despite these recent findings, a major unanswered question is how do gut bacteria dictate or shape extraintestinal immune responses that promote tumor killing in distant sites. The potential immunologic influence or impact of gut bacteria on cancers that arise in the gastrointestinal tract (e.g. colorectal, pancreatic, and liver cancer) appears to be more obvious, given the close proximity of gut bacteria, immune cells, and the tumor. How gut bacteria modulate immune responses against extraintestinal tumors, such as melanoma or lung cancer, is unclear.

Here, we used a preclinical melanoma model and anti-PD-1/CTLA-4 therapy to show that ICT induces translocation of specific gut bacteria into secondary lymphoid organs and tumors, which activates dendritic cells (DC) and primes anti-tumor T cell responses. Gut bacteria translocation into mesenteric lymph nodes (MLN) is essential for optimal anti-tumor T cell responses in the tumor draining lymph node (TDLN) and tumor. DCs are critical not only for the gut bacteria-dependent immune augmentation of ICT but also for facilitating gut bacteria translocation into MLN. Furthermore, we find that ICT induces MLN remodeling, which can facilitate further dissemination of bacteria beyond the intestinal mucosal firewall. Finally, antibiotic depletion of endogenous gut bacteria results in decreased gut bacteria translocation into MLN and TDLN, diminished DC activation and effector T cell responses, thus causing attenuated ICT efficacy. Together, our studies reveal a critical mechanism in which ICT aided by DCs causes gut bacteria translocation of specific immunogenic taxa into secondary lymphoid organs, resulting in optimal priming of anti-tumor immune responses effective against extraintestinal tumors.

## RESULTS

### Immune checkpoint inhibitor therapy induces gut bacteria translocation into secondary lymphoid organs and tumor

We first sought to determine whether ICT, in the absence or presence of tumor, could induce gut bacteria translocation into secondary lymphoid organs. We administered ICT (combined anti-PD-1 and anti-CTLA-4 monoclonal antibody treatment) to mice with or without B16-F10 melanoma tumors and assessed bacterial translocation by culturing mesenteric lymph node (MLN) homogenates on various selective media under anaerobic conditions (fig. S1A). Indeed, mice receiving ICT had a greater number of cultured bacteria from MLNs (fig. S1B), including taxa previously reported to be associated with positive ICT response or implicated in host immune-anti cancer responses: *Bifidobacterium pseudolongum* (9), Bifidobacterium breve, Bifidobacterium longum, Bifidobacterium adolescentis (13), *Bacteroidetes thetaiotamicron* (7, 24), Enterococcus faecalis (22, 24), and *Lactobacillus spp*. (24) (fig. S1C). Interestingly, the presence of melanoma tumor alone did not result in a dramatic increase of cultured bacteria from MLNs whereas the administration of ICT, regardless of tumor presence, resulted in a significant increase in the number of bacteria cultured from MLN tissue on YCFA and CME0151 agar (YCFA, p=0.0037; CME0151, p=0.0006; fig. S1B).

**Fig. 1.**
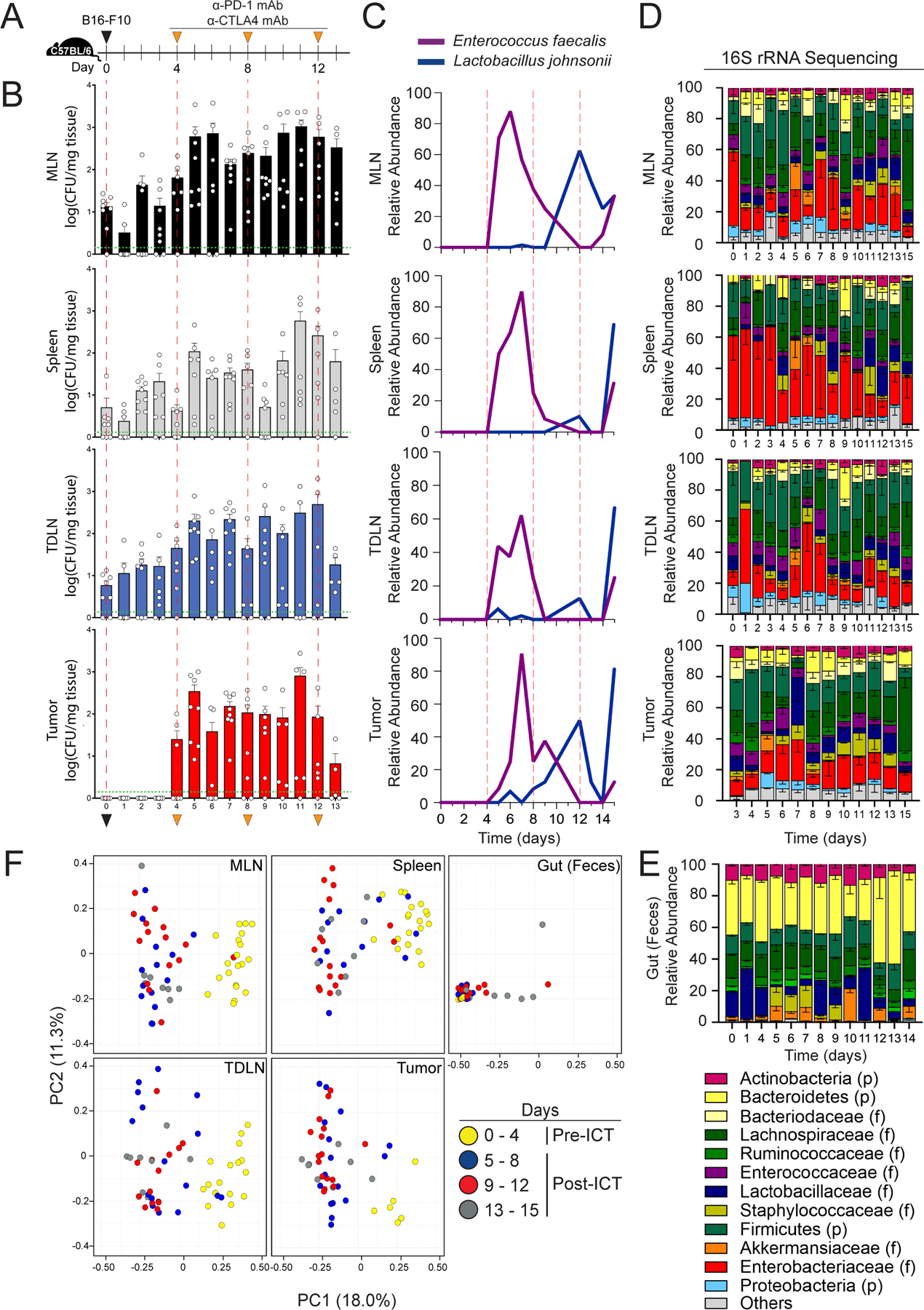
Immune Checkpoint Inhibitor Therapy (ICT) Induces Gut Microbiota Translocation into Secondary Lymphoid Organs and Tumor (**A**) Schematic diagram of the strategy used to assess the temporal dynamics of bacterial translocation into secondary lymphoid organs and tumor in C57BL/6 mice (female, 6–8 wks, Jackson) bearing B16-F10 melanoma tumors and receiving ICT (anti-PD-1 and anti-CTLA-4 mAb, 200μg). (**B)** Cultured bacterial levels in MLN, Spleen, TDLN, and Tumor. Tissue homogenates were serially diluted, plated on BHI/Blood agar, and cultured at 37°C under anaerobic conditions for 24–72 hours. n=3–4 per time point per experiment. Two experiments were performed for a final sample size of n=6–8 per group. Points represent values from individual mice. Bars represent the mean ± SEM. Green dotted lines represent the limit of detection. (**C**) Relative abundance of cultured bacteria from secondary lymphoid organs and tumor, as determined by full length (V1-V9) 16S rRNA amplicon sequencing (Sanger). Sequences were entered into the NCBI standard nucleotide Basic Local Alignment Search (BLAST) tool utilizing the rRNA/ITS databases. Bacterial species identification was ascertained from BLASTN results with the highest Total Score, with percent identity score >95% and E value <0.01. (**D-F**) 16sRNA gene sequencing (V4 region) of tissue gDNA isolated from mice as detailed in fig. 1A. (**D**) Relative abundance of microbiota in MLN, spleen, TDLN, as determined by 16S rRNA sequencing. (**E**) Relative abundance of microbiota in the gut (feces), as determined by 16S rRNA sequencing. (**F**) Principal coordinate analysis of tissue and gut 16S rRNA sequencing data, weighted and normalized by Bray-Curtis distances. The proportion of variance accounted by each principal component is indicated

We then asked whether there was a distinct temporal pattern of bacterial translocation throughout the course of ICT treatment. To address this question, we implanted B16-F10 tumors in a cohort of mice simultaneously and then proceeded with ICT treatment (fig. 1A). On each day, we selected mice (n=6–8) from different cages and harvested MLN, spleen, TDLN (defined as the right inguinal lymph node as tumors were implanted in the right flank), and tumor. Tissue microbiomes were assessed by both culturing of tissue homogenates and 16S rRNA sequencing of tissue gDNA. Notably, bacterial translocation was present but limited (< mean of 65 CFU/mg tissue) for all tissue types before the first dose of ICT (day 4) (fig. 1B). After initiation of ICT, bacterial translocation was readily detected in each tissue type (but to the greatest magnitude in MLN) and persisted throughout the course of ICT (fig. 1B). These data suggest that ICT induces gut bacteria translocation into secondary lymphoid tissues and tumor.

Upon taxonomic identification of cultured bacteria, two dominant taxa, *Enterococcus faecalis* and *Lactobacillus johnsonii*, and distinct temporal pattern of gut microbiota translocation were noted (fig. 1C). *E. faecalis* was the most abundant gut microbiota translocator during the early phases of ICT treatment (between doses 1 and 2) while *L. johnsonii* exhibited dominance after the second dose of ICT (fig. 1C). Other notable cultured taxa include *Akkermansia muciniphila* (12) and *Bacteroides thetaiotaomicron* (14), both of which have been shown to augment ICT responses in preclinical models (fig. S1 and S2).

The ability to successfully culture gut microbiota is highly variable, thus we sought to further characterize the secondary lymphoid organ and tumor microbiomes using 16S rRNA gene sequencing (fig. 1D–F, fig. S3–5). Using multidimensional scaling of the 16S rRNA data and factoring both tissue type and the time of sample collection, we noted that the gut microbiome taxonomic composition remained steady over the course of ICT (exhibited by the tight clustering of gut microbiome samples, diamonds, in the lower left-hand corner and demarcated with an ellipse, fig. S3). In contrast, while lymphoid tissue and tumor samples collected prior to the first dose of ICT (days 0–4) also grouped together regardless of tissue type (fig. 1F and S3, right side, various tissue type samples all in yellow), notable taxonomic shifts occur after ICT initiation (fig. 1F and S3). By using singular value decomposition interpreted visually as a linear biplot (25), we identified six taxa as key drivers of taxonomic compositional changes in secondary lymphoid organs and tumor at later time points (days 5–15): *Enterococcus*, Lachnospiraceae, and *Escherichia/Shigella* accounting for the majority of change, with *Lactobacillus, Muribaculaceae*, and *Turibacter* contributing as well (fig S3, demarcated as labeled arrows). When utilizing another well-established microbiome taxonomic differential abundance analysis tool (linear discriminant analysis effect size, LEfSe), we confirmed that *Enterococcus*, Lachnospiraceae (*Blautia* spp.), and *Shigella* were significantly enriched in tumor and secondary lymphoid organ tissues compared to the gut (>2 log-fold increase in linear discriminate analysis, LDA, score and P<0.05, fig. S4). Interestingly, bacterial taxa abundant in the gut (e.g. Bacteroidetes) were not necessarily highly abundant in the secondary lymphoid organs or tumors and *vice versa* (e.g. low abundance of Enterobacteriaceae in the gut, but high relative abundance in tissues) (fig. 1D and E). Finally, overall levels of bacteria (as determined by bacterial group qPCR of the same gDNA samples used for 16S rRNA sequencing shown in fig. 1D) showed a comparable variation over time to that observed with the cultured microbiota abundance (fig 1B), with an initial increase followed by sustained bacterial levels throughout ICT administration (fig. S5). Taken together, these results suggest that ICT induces translocation of specific gut bacteria, which are not necessarily abundant in the gut, into secondary lymphoid organs and tumor.

### Highly abundant bacterial translocators into secondary lymphoid organs activate DCs and induce anti-tumor effector T cell responses

**Figure 2.**
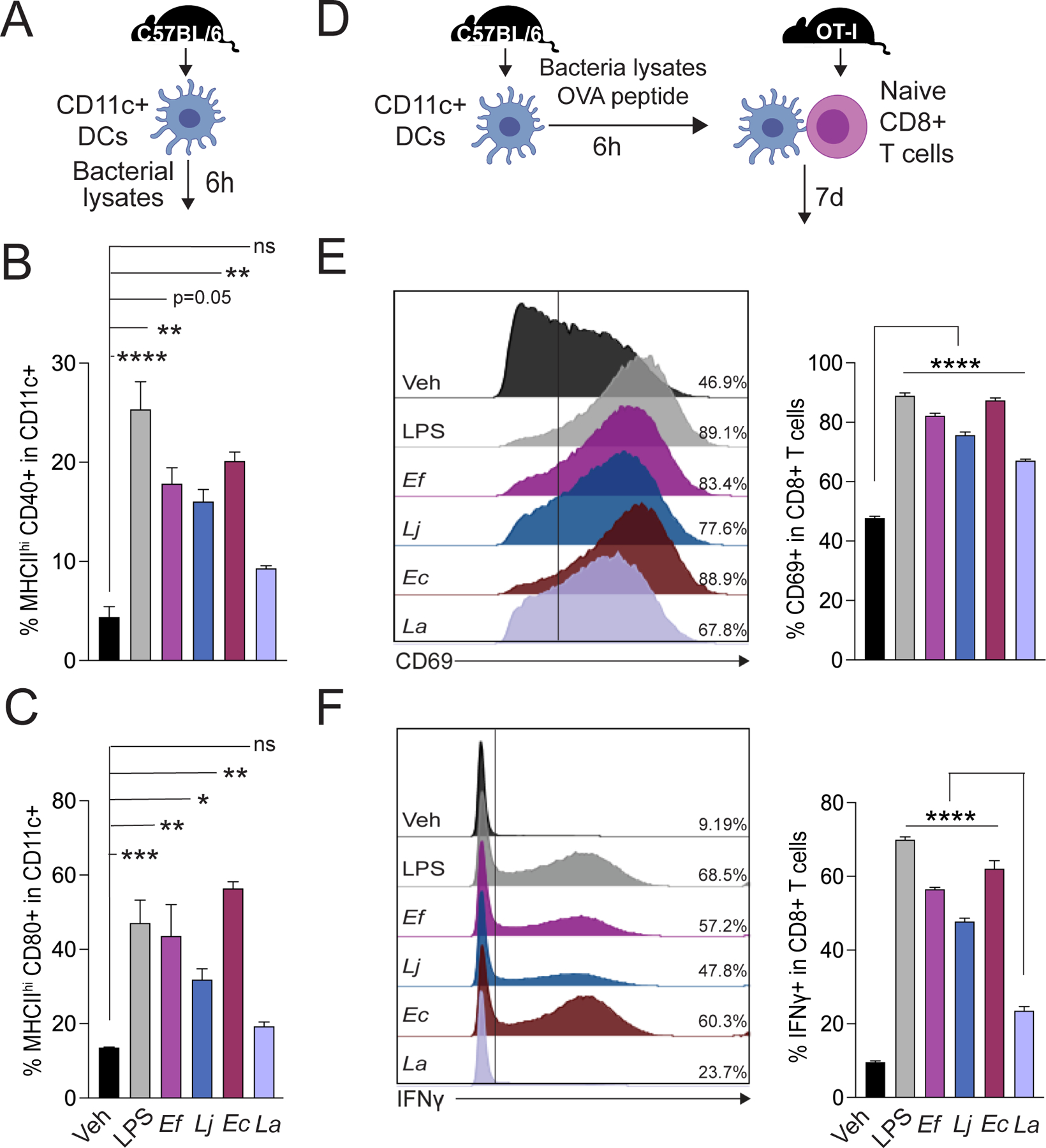
Highly abundant microbiota translocators into secondary lymphoid organs activate DCs and induce anti-tumor effector T cell responses (**A**) Schematic overview of the protocol used to assess the dendritic cell (DC)-activating potential of various gut bacteria. CD11c+ DCs were isolated from the spleen of C57BL6/J mice (female, 6–8 wks, Jackson) bearing B16-FLT3L tumors. Isolated DCs were stimulated with vehicle (PBS), *Escherichia coli* LPS, *Enterococcus faecalis* (*Ef*), *Lactobacillus johnsonii* (*Lj*), *Escherichia coli* (*Ec*), or *Lactobacillus acidophilus* (La) lysates for 6 hours. DCs were then analyzed by flow cytometry. Proportion of (**B**) MHC-II high, CD40+ cells and (**C**) MHC-II high, CD80+ cells among CD11c+ DCs. (**D**) Schematic overview of the protocol used to assess T cell activating and priming potential of DCs stimulated with different gut bacteria. CD11c+ DCs were pulsed with OVA 257–264 peptide and bacterial lysates for 6h. Stimulated DCs were then co-cultured with naïve CD8+ T cells isolated from age- and sex-matched OT-I mice for 7d. Surface expression of T cell activation marker CD69 and intracellular interferon-γ (IFN-γ) were quantified by flow cytometry. Proportion of (**E**) CD69+ and (**F**) IFN-γ+ cells among CD8+ T cells. Bars represent the mean ± SEM. All assays were performed in triplicate. Statistical analysis by Mann-Whitney test. *P<0.05, **P<0.01, ***P<0.001, ****P<0.0001.

We then asked whether the most abundant translocated bacteria identified by culturing and sequencing (specifically *Enterococcus* spp., *Lactobacillus johnsonii*, and Enterobacteriaceae (fig. 1)) induce distinct immune responses to facilitate anti-cancer immunity. Translocated bacteria or bacterial components can provide a diverse array of pathogen-associated molecular patterns (PAMPs) capable of agonizing various pattern recognition receptors (PRRs) and thus activating innate immune responses (26, 27). Thus, we hypothesized that translocated gut bacterial components activate dendritic cells (DCs) that consequently drive tailored T cell immunity (28). Indeed, mouse DCs stimulated with *Enterococcus faecalis, Lactobacillus johnsonii*, and Enterobacteriaceae member *Escherichia coli* (fig. 2A) showed a marked increase in the surface expression of MHCII and co-stimulatory receptors, CD40, CD80 and CD86 (fig. 2B and C, fig. S6), whereas stimulation with *Lactobacillus acidophilus*, a common gut microbiota commensal and over-the-counter probiotic constituent, did not significantly activate DCs. The decreased immunogenic activity of *Lactobacillus acidophilus* has been previously described and may be due to immunomodulatory CpG motifs (29). We then investigated whether these gut microbiota translocators had a differential capacity to prime naïve T cells. We used an antigen-restricted DC-T cell priming system, MHC class-I-restricted, ovalbumin-specific, CD8+ T cells from OT-I TCR transgenic mice, and measured the activation and functional phenotypes of T cells after the co-culture with DCs (fig. 2D) (28). While OT-I CD8+ T cells primed with DCs pulsed with all bacteria showed significantly higher activation (p<0.0001, fig. 2E), only DCs pulsed with highly abundant microbiota translocators *E. faecalis*, *L. johnsonii*, and *E. coli* induced significantly greater interferon-γ (IFN-γ) production in CD8+ T cells compared to the non-stimulated and *L. acidophilus* groups (p<0.0001, fig. 2F). These data are consistent with prior published studies highlighting the importance of DCs in anti-tumor immunity in the setting of ICT (30–33), and also are concordant with our prior work highlighting the differential capacity of specific gut bacteria in driving specific T cell immune responses (e.g. Th1 vs Th17, etc.) (28).

### Mesenteric lymph nodes modulate gut bacteria-dependent anti-tumor priming responses in the tumor-draining lymph node and tumor

We hypothesized that gut bacteria translocation into secondary lymphoid organs, particularly the mesenteric lymph nodes (MLN), is critical for shaping extra-intestinal anti-tumor responses in the tumor draining lymph node (TDLN) and the tumor. As a first step, we implanted melanoma tumors into lymphotoxin-〈 knockout mice (LTA KO) that lack secondary lymphoid organs, including MLN, inguinal lymph nodes, and Peyer’s patches (34) followed by ICT administration. Indeed, LTA KO mice had a diminished response to ICT, despite having intact gut microbiota, compared to co-housed wild-type controls (fig. S7). Of note, while LTA KO mice have normal quantitative and qualitative immune functions in general (34), a number of immune defects such as lack of follicular dendritic cells, altered splenic morphology accompanied by changes in T and B cell content, and increased peripheral B cell numbers may have directly affected the ICT efficacy observed here. To further investigate the importance of secondary lymphoid organs in modulating gut bacteria dependent anti-tumor immune responses, we administered FTY-720 (fingolimod), a sphingosine-1-phosphate receptor modulator that sequesters lymphocytes in lymph nodes, to mice bearing melanoma tumors and receiving ICT. Mice receiving FTY-720 and ICT had larger tumor volumes compared to ICT only controls, despite having intact gut microbiota and intact lymph nodes (fig. S8). These data suggest that secondary lymphoid organs and lymphocyte egress from these lymphoid tissues are critical for optimal gut bacteria-induced anti-cancer responses in the setting of ICT.

**Figure 3.**
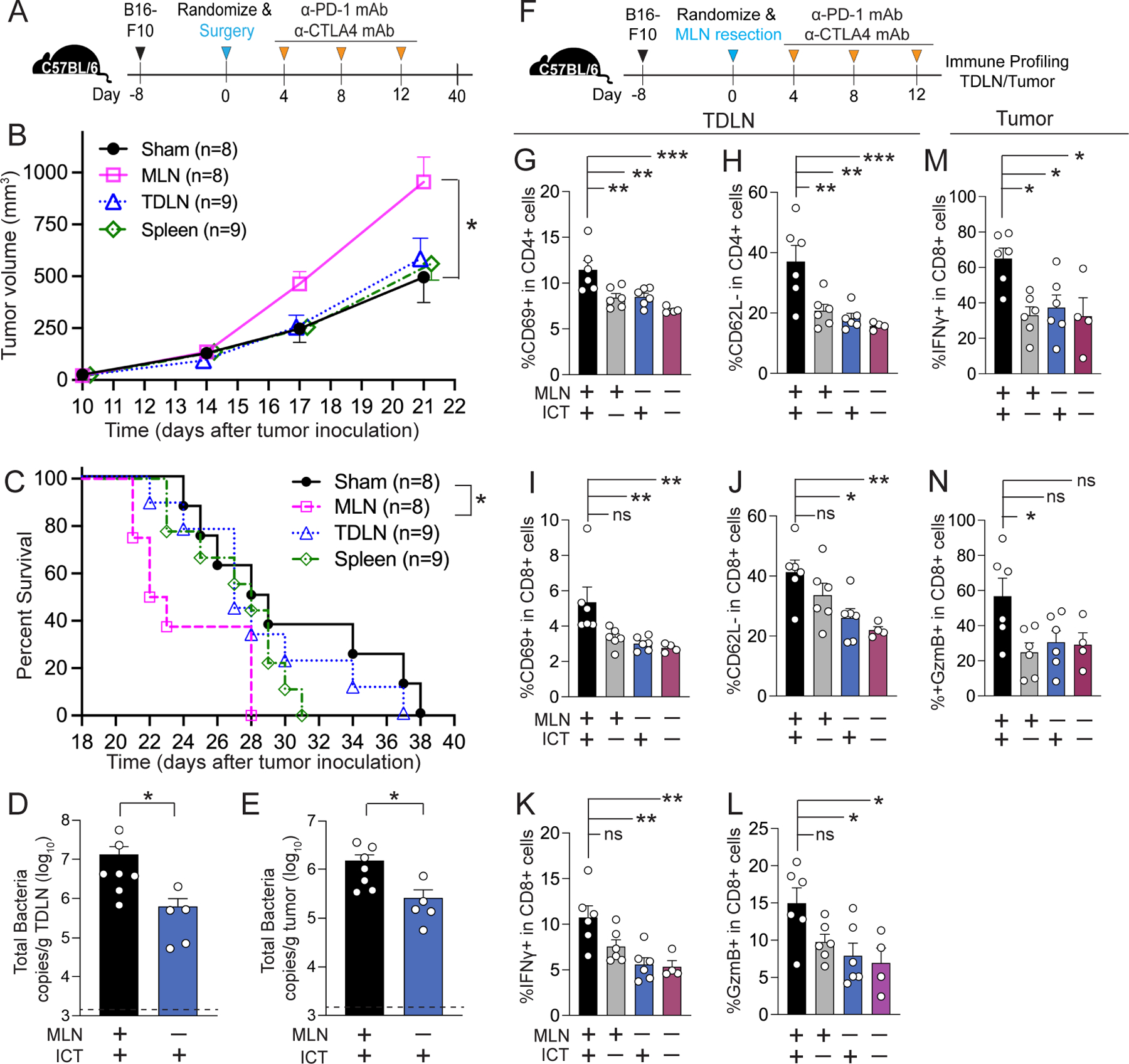
Mesenteric lymph nodes modulate gut microbiota-dependent anti-tumor priming responses in the tumor-draining lymph node and tumor (**A**) Schematic overview of the protocol used to assess the impact of surgical resection of secondary lymphoid organs or control (sham surgery involving longitudinal abdominal incision only) on ICT efficacy. C57BL/6 mice (female, 6–8 wks, Jackson) were inoculated with 1 × 10^5^ B16-F10 cells subcutaneously in the right flank. Mice with comparable tumor volumes were randomized to receive surgery. 200μg anti-PD-1 and 200μg anti-CTLA-4 mAb (ICT) were injected intraperitoneally on days 4, 8, and 12 post-surgery. n=8–9 per group. (**B**) Tumor volume and (**C**) survival of tumor-bearing mice as detailed in fig 3A. Total bacterial load in tumor-draining lymph node (TDLN) (**D**) and tumor (**E**) of mice ± MLN ± ICT, as determined by bacterial group (Eubacteria, all bacteria) quantitative-PCR (qPCR) of tissue gDNA collected from mice as detailed in fig. 3A. **(D-E)** Dotted lines represent the limit of detection. n=5–7 per group. (**F**) Schematic overview of the protocol used to assess the impact of MLN removal on the CD4+ and CD8+ T cell immune responses in TDLN and tumor of B16-F10 tumor-bearing mice receiving ICT. n=4–6 per group (**G-L**) Quantification of CD4+ and CD8+ T cell immune response in the TDLN of mice ± MLN by flow cytometry. The proportion of (**G, I**) activated (CD69+) and (**H, J**) effector (CD62L-) T cells among CD4+ T cells and CD8+ T cells respectively. The proportion of (**K**) IFN-γ and (**L**) granzyme B (GzmB) producing cells among CD8+ T cells. (**M-N**) Quantification of CD8+ T cell immune response in the tumor of mice ± MLN by flow cytometry. The proportion of (**M**) IFN-γ and (**N**) granzyme B (GzmB) producing cells among CD8+ T cells. For all experiments, points represent results from individual animals. n=4–6 per group. Bars represent the mean ± SEM. Statistical analysis by Mann-Whitney test. *P<0.05, **P<0.01, ***P<0.001.

We then asked whether specific secondary lymphoid organs had a differential capacity to modulate extra-intestinal anti-tumor responses. Hence, we surgically resected different secondary lymphoid organs (MLN, TDLN, or spleen) from mice bearing melanoma tumors with intact gut microbiota and then administered ICT (fig. 3A). Interestingly, only mice with MLN resection had significantly larger tumor volumes compared to control mice (receiving sham surgery) (p= 0.0144, fig. 3B). Similarly, while all surgical groups exhibited decreased survival time, only mice lacking MLN had significantly decreased survival compared to sham surgery control mice (p= 0.025, log-rank test, fig 3C). Further, MLN removal led to a significant decrease in bacterial load in TDLN (p=0.0101, fig. 3D) and tumor (p=0.0177, fig 3E), suggesting that MLN may play a role as a conduit of intact bacteria or gut bacteria-derived components from the gut to extra-intestinal sites, such as TDLN and/or tumor.

As surgical resection of MLN attenuated response to ICT, we further investigated the impact of MLN removal on anti-cancer immune responses (fig. 3F). The number of immune cells (CD45+ cells) in TDLNs was significantly lower in mice lacking MLN (p=0.0058, fig. S9A). MLN resection resulted in a significant decrease in CD11c+ dendritic cells (p=0.0009, fig. S9B; fig. S9C) as well as activated effector CD4+ and CD8+ T cell subsets (CD69+ and CD62L-) in TDLNs (fig. 3G–J, fig. S10). Indeed, the proportion of TDLN CD8+ T cells producing IFN-γ (fig. 3K, fig. S11) and Granzyme B (fig. 3L, fig. S11) was markedly decreased in mice lacking MLN. Further, MLN resection resulted in significantly lower leukocyte cell infiltration into the tumor (p=0.039, fig. S12) and a concomitant reduction in the proportion of tumor CD8+ T cells producing IFN-γ (fig. 3M, fig. S13) and Granzyme B (fig. 3N, fig. S13). These data suggest that MLNs likely play a critical role in mediating the gut bacteria-dependent extra-intestinal anti-tumor immune effects observed with ICT, perhaps by functioning as a gateway by which gut bacteria-induced immunogenic signals are delivered to more distal body niches.

### Dendritic cells facilitate gut bacteria translocation into secondary lymphoid organs

We then investigated potential mechanisms by which gut microbiota translocation into secondary lymphoid organs was facilitated in the context of ICT. While combination anti-PD-1 and anti-CTLA-4 therapy has been shown to be clinically effective against various tumor types, the high incidence of developing immune-related adverse events, such as colitis, limits widespread use, particularly in older patients with multiple medical comorbidities (18). Thus, we explored the possibility that ICT-induced gut inflammation could lead to a disruption of intestinal epithelial barrier function thus promoting gut microbiota translocation (35). We orally administered fluorescein isothiocyanate (FITC)–dextran to measure gut permeability in tumor-bearing mice treated with or without ICT (fig. S14A). ICT did not increase the serum FITC-dextran levels during the course of ICT (fig. S14B). Further, ICT did not alter the mRNA expression of ZO-1, an epithelial tight junction protein, in the intestine of mice (fig. S14C). These data suggest that increased gut permeability may not be a major mechanism of ICT-induced gut bacteria translocation in this preclinical model.

**Figure 4.**
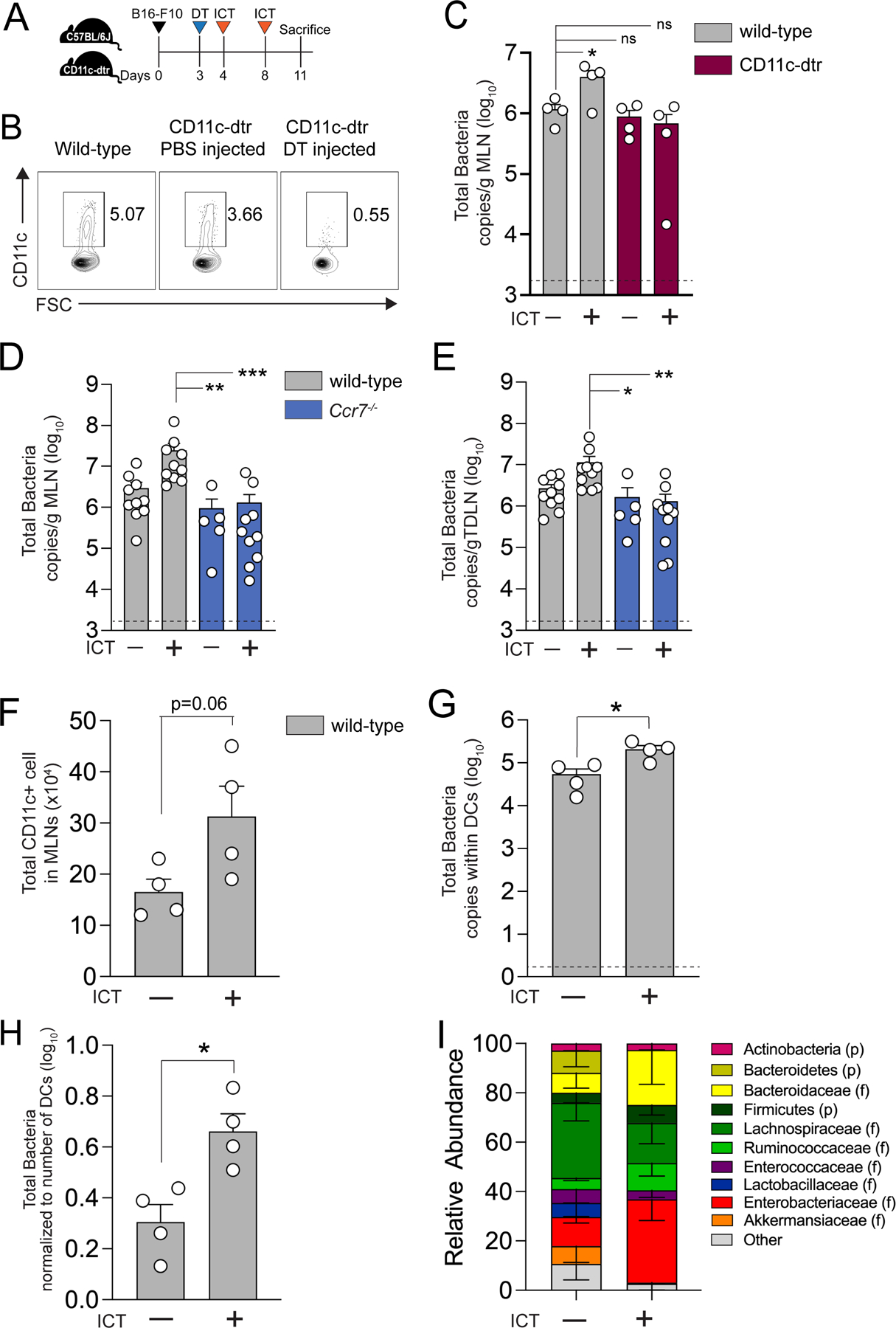
DCs facilitate ICT-induced gut microbiota translocation into secondary lymphoid organs (**A**) Schematic overview of the protocol used to assess the impact of dendritic cell (DC) depletion on ICT-induced microbial translocation into MLN. CD11c-dtr mice (female, 6–8 wks, Jackson) were injected with 100 ng diphtheria toxin (DT) intraperitoneally on day 3 post tumor implantation to deplete CD11c+ DCs. Wild-type C57BL/6 and DT-treated CD11c-dtr mice were injected with 200μg anti-PD-1 and 200μg anti-CTLA-4 mAb intraperitoneally on days 4 and 8 post tumor implantation. (**B**) Representative flow cytometry plot of CD11c+ subsets. (**C**) Bacterial load of MLN in wild-type or CD11c-dtr DC-depleted mice ± ICT, as determined by bacterial group (Eubacteria, all bacteria) quantitative-PCR of MLN gDNA collected from mice as detailed in fig. 4A. n=4 per group. Bacterial load in (**D**) MLN and (**E**) TDLN recovered from wild-type (C57BL/6J) and *Ccr7*^−/−^ mice ± ICT. n=10 per group. **(C-E)** Dotted lines represent the limit of detection. (**F-H**) Bacterial load in DCs. CD11c+ DCs were isolated from MLN of C57BL/6 mice (female, 6–8 wks, Jackson) bearing B16-F10 melanoma tumors ± ICT. gDNA was isolated from DCs (from 5 mice pooled into one sample). Bacteria load was determined by bacterial group (Eubacteria, all bacteria) quantitative-PCR of DC gDNA. (**F**) Number of CD11c+ DCs isolated from MLN of wild-type mice ± ICT. (**G**) Quantification of bacterial load within DCs isolated from MLN of mice ± ICT. Dotted line represents the limit of detection. (**H**) Quantification of bacterial load within DCs normalized to the total number of DCs. (**I**) Relative abundance of microbiota in dendritic cells, as determined by 16S rRNA sequencing. For (**D, E**), points represent results from individual animals. For (**F-H**), each point represents a single biological replicate with DCs pooled from 5 mice. Total of n=20 per group. Bars represent the mean ± SEM. Statistical analysis by Mann-Whitney test. *P<0.05, **P<0.01, ***P<0.001.

We leveraged the knowledge that DCs can retain live commensal gut bacteria for several days and carry or traffic microbiota to MLNs (36) and hypothesized that the ICT-induced gut bacteria translocation could be a DC-dependent phenomenon. To test this hypothesis, we utilized CD11c-dtr transgenic mice, in which the CD11c promoter (*ltgax*) directs the expression of a diphtheria toxin receptor (dtr), and administration of diphtheria toxin allows for the depletion of DC populations (37). Indeed, a single dose of intraperitoneal diphtheria toxin (DT) injection (100ng) efficiently depleted CD11c+ DC populations from MLNs (fig. 4A and B). We then measured the total bacterial load in MLNs of wild-type and CD11c-dtr mice (as determined by quantifying genomic copies of 16s ribosomal RNA gene within the total genomic DNA extracted from MLNs of mice treated with or without ICT and DT). We observed that ICT induces bacterial translocation into MLNs in wild-type mice but not in CD11c-dtr mice treated with DT (fig. 4C). This result suggests a potential key role for DCs in ICT-induced bacterial translocation into secondary lymphoid organs.

CCR7 has been shown to be important for the entry of lymphocytes and DCs into secondary lymphoid organs (38, 39). Hence, we next assessed the role of CCR7 in ICT-induced gut microbiota translocation into secondary lymphoid organs. Wild-type C57BL/6J and *Ccr7*^−/−^ mice were implanted with melanoma tumors and treated with ICT. Consistent with our previous results, the increase in ICT-induced bacterial translocation into the MLNs and TDLNs was not observed in *Ccr7*^−/−^ mice (fig. 4D and E). Prior studies have reported similar findings that augmenting inflammation in the gastrointestinal tract (e.g. *Rnf5*^−/−^ mice (40) or *Salmonella* infection (39)) increases DC mobilization into MLNs and TDLNs in a CCR7-dependent manner.

Anti-PD-1 and/or anti-CTLA-4 blockade therapy promotes DC activation and migration in a T cell dependent manner (41–43). Thus, we hypothesized that ICT promotes pro-inflammatory cytokine secretion by T cells within the GI tract, thereby further inducing activation and migration of mucosal DCs. To test this hypothesis, we first tested the direct stimulatory impact of ICT-induced pro-inflammatory cytokines or anti-PD-1/CTLA-4 mAbs on CD11c+ DCs *ex vivo*. Consistent with prior published reports (44–46), pro-inflammatory cytokines such as TNF-α, IFN-γ, and IL-1β, but not ICT monoclonal antibodies, induced significant upregulation of surface expression of costimulatory receptors (CD80 and CD86) and a chemokine receptor (CCR7) on DCs (p < 0.0001, fig. S15). We then tested whether ICT induces production of pro-inflammatory cytokines and activation of different DC subsets in the murine GI tract. Indeed, we observed that mice treated with ICT had increased expression of the pro-inflammatory genes *Tnfa* and *Il1b* in the small intestine and colon (p= 0.0262, fig. S16). Further, the number of DCs present in the lamina propria (LP) and the DC surface expression of MHC II were higher in ICT-treated mice compared to isotype treated controls (fig. S17A). The number of conventional DC1 (CD103+CD11b-CD11c+) and conventional DC2 (CD103-CD11b+CD11c+) cells expressing high levels of CD80 and MHC II molecules was also higher in ICT treated mice compared to isotype controls (fig. S17B and C) (47). These collective results suggest that ICT induced gut inflammation may play a role in promoting DC mobilization, and thus trafficking of gut bacteria into secondary lymphoid organs in a CCR7-dependent fashion.

Finally, to further confirm the ability of gut bacteria to reside within DCs (36), we isolated CD11c+ DCs from the MLN of wild-type mice treated with or without ICT and measured total bacterial load. Not only was the total number of CD11c+ cells higher in MLNs of mice treated with ICT (fig. 4F), but the total bacterial load was significantly higher within the DCs isolated from MLNs in mice treated with ICT as well (p= 0.0186, fig. 4G). Even when normalizing to the number of DCs recovered, the total bacterial load per DC was higher in mice treated with ICT (fig. 4H). To determine the identity of the bacteria trafficked by DCs, we performed 16S rRNA sequencing on gDNA extracted from MLN DCs in melanoma-bearing mice treated with or without ICT. The identified taxa in DCs (fig. 4I) were commensurate with the tissue and tumor microbiomes shown previously (fig. 1D). Interestingly, Enterobacteriaceae were significantly enriched in MLN DCs in mice treated with ICT (p= 0.049, fig. S18), findings which may partially explain our prior observation that Enterobacteriaceae (*Shigella*) was significantly enriched in tumor and secondary lymphoid organ tissues of mice treated with ICT despite low relative abundance in the gut (fig. 1E and S3). Collectively, these results suggest DCs play an integral role in ICT-induced gut bacteria translocation mediated anti-tumor immune effects.

### ICT induces lymphangiogenesis and dilation of high endothelial venules in MLN

**Figure 5.**
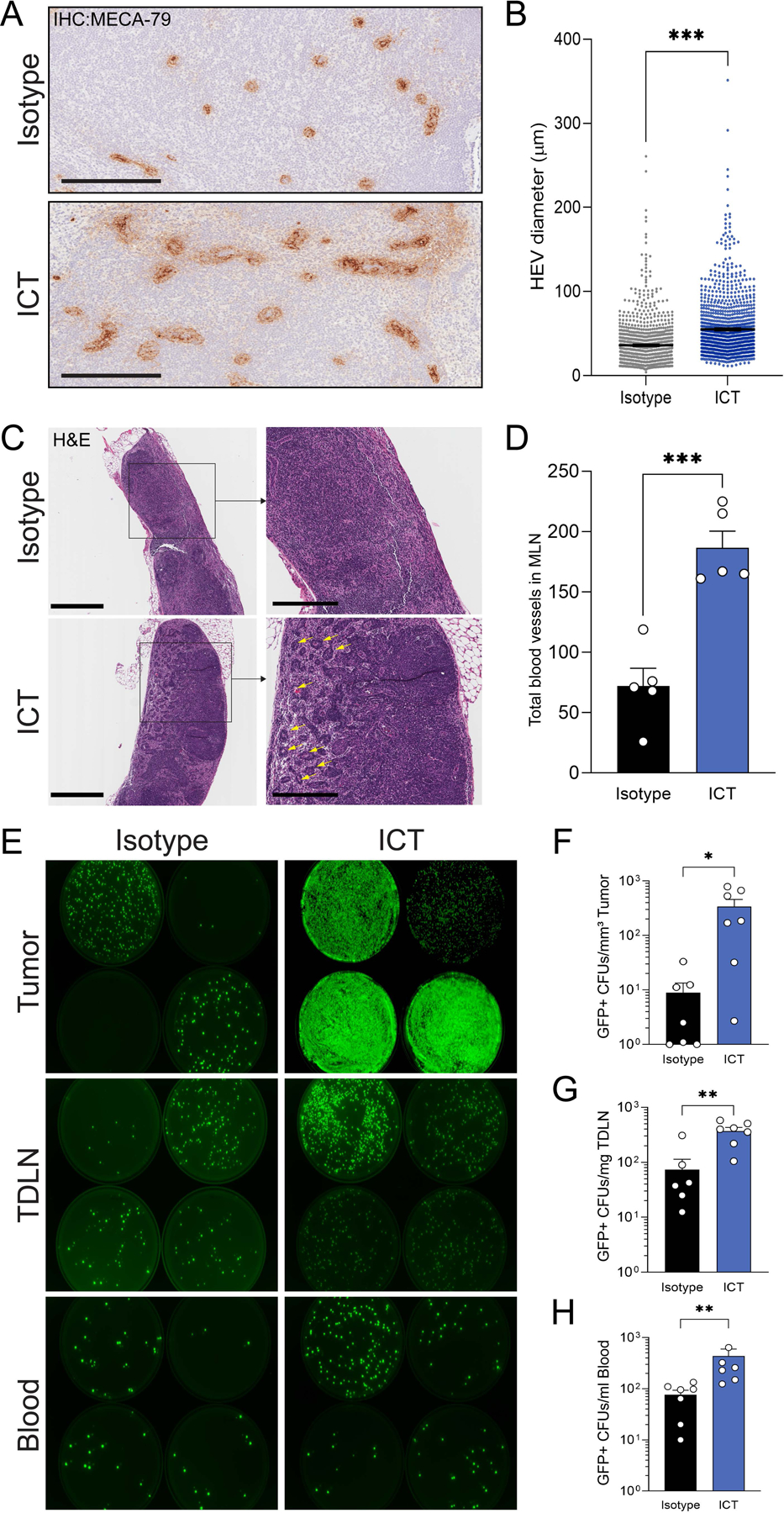
ICT induces MLN remodeling **(A-D)** C57BL/6 mice (female, 6–8 wks, Jackson) were inoculated with 1 × 10^5^ B16-F10 cells subcutaneously in the right flank. 200μg anti-PD-1 and 200μg anti-CTLA-4 mAb (ICT) or isotype antibodies were injected intraperitoneally × 3. MLN tissue was fixed and processed for high endothelial venule marker MECA-79 immunohistochemistry staining (**A**, **B**) and hematoxylin and eosin (H&E) staining (**C**, **D**). (**A**) Representative images of MECA-79 staining. Scale bar = 200μm. (**B**) Quantification of high endothelial venules (HEVs) diameter in MLN from mice bearing B16-F10 tumors and treated with or without ICT. n=5 mice per group. Statistical analysis by t-test. ***P<0.001 (**C**) Representative image of H&E staining. Scale bar = 600μm (Left panels), 300μm (Right panels). Yellow arrows indicate blood vessels in the medullary space (ms) (**D**) Quantification of total number of blood vessels in the MLN medullary space of mice bearing B16-F10 tumors treated with or without ICT. n=5 mice per group. Statistical analysis by t-test. ***P<0.001 (**E-H**) C57BL/6 mice (female, 6–8 wks, Jackson) were inoculated with 1 × 10^5^ B16-F10 cells subcutaneously in the right flank. Mice with comparable tumor volumes were randomized before the ICT. 3 doses of ICT or isotype controls were injected intraperitoneally (n=6–7 per group). 1 × 10^7^ GFP+ *E. coli* were injected directly into the MLN. Tumor, TDLN and blood was collected 24 hours post *E. coli* injection. Tissue homogenates and blood were spread on TSA-Kanamycin Agar plates. GFP+ colonies were enumerated after 24 hours of incubation at 37°C. (**E**) Representative image of GFP+ colonies. Quantification of GFP+ CFUs in (**F**) tumor, (**G**) TDLN and (**H**) Blood. Bars represent the mean ± SEM. Statistical analysis by t-test. *P<0.05, **P<0.01, ***P<0.001.

Tumor development has been associated with distinct anatomical changes in the TDLN, a phenomenon termed ‘lymph node remodeling,’ characterized by 1) the expansion of lymphatic sinuses and proliferation of lymphatic endothelial cells (lymphangiogenesis) and 2) dilation of high endothelial venules (HEVs), and these changes facilitate the dissemination of cancer cells from draining lymph nodes to distal locations (48, 49). Further, lymph node remodeling has also been reported as a common feature of inflamed or immune-reactive lymph nodes (50–52). Thus, we investigated whether ICT-induced inflammation could induce MLN remodeling by treating mice with ICT or isotype controls and measuring key features of lymph node remodeling, (lymphangiogenesis and HEV dilation) via immunohistochemistry and microscopy. Indeed, ICT-treated animals showed a significant enlargement of HEVs in the MLN compared to isotype-treated counterparts (p < 0.001, fig. 5A and B). Further, we observed an increase in the number of blood vessels (~2.6 fold increase) and expansion of the medullary sinus within the MLN of ICT-treated animals compared to isotype controls, suggesting that ICT promotes a significant lymphangiogenesis within the ICT-treated MLN (p < 0.001, fig. 5C and D). These results are consistent with prior data showing that endothelial cell expansion in lymph nodes is induced by DCs (53) and our data showing that ICT induces DC recruitment into MLN (fig. 4F). In sum, ICT induces remodeling of MLN, dramatically increasing lymphangiogenesis and the HEV dilation within the MLN.

We then asked whether the ICT-induced remodeling affects the antigen restricting capacity of MLN, a concept commonly referred to as “the mucosal firewall” (36, 54). We performed intra-MLN injections of green fluorescent protein-expressing bacteria (GFP+ *E. coli*) following preconditioning with ICT and enumerated GFP+ *E. coli* colonies cultured from tumor, TDLN and blood 24 h after bacterial injection. We detected significantly higher GFP+ *E. coli* burden in the tumor (p=0.0176), TDLN (p=0.0017), and blood (p=0.0012) from mice treated with ICT compared to isotype-treated controls (fig. 5E–H). Finally, in order to determine if this observed phenomenon is DC-dependent, we measured the degree of bacterial dissemination from MLN to tumor in wild type C57BL/6 or DC depleted mice (CD11c-dtr mice treated with diphtheria toxin). There was no significant difference in the number of GFP+ bacteria in the tumors of wild type and DC-depleted mice suggesting that bacterial dissemination from MLN to tumor may not be dependent on DCs (Fig. S19). Together, these results suggest that ICT-induced lymphangiogenesis and dilation of HEVs in MLN may facilitate bacterial translocation from the MLN to distal locations such as tumor and TDLN.

### Antibiotic treatment results in decreased gut bacteria translocation into MLN, decreased polyfunctional CD8+ T cell effector responses, and diminished ICT efficacy

Antibiotic exposure has been associated with inferior clinical outcomes in cancer patients receiving ICT (6, 12, 55–57). Germ-free mice and mice pre-treated with antibiotics are less responsive to ICT (13, 14). Yet, the mechanisms by which antibiotic treatment attenuates effectiveness of ICT are unclear. We posited that antibiotic treatment would reduce overall levels of gut bacteria, thus reducing gut bacteria translocation into secondary lymphoid organs and tumor, thereby mitigating gut microbiota-induced immune activation and ultimately attenuating ICT efficacy.

**Figure 6.**
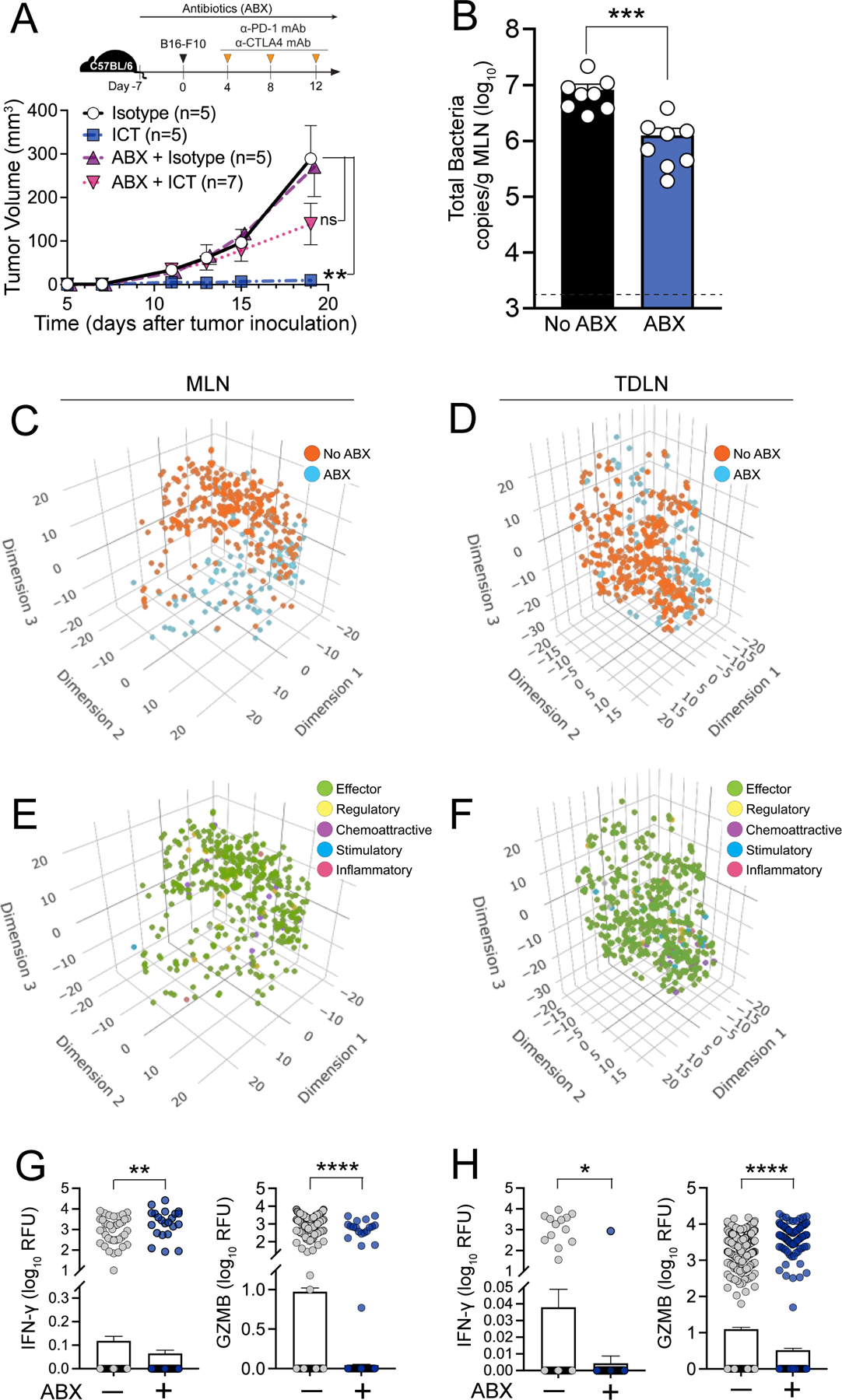
Antibiotic treatment results in decreased gut microbiota translocation into MLN, decreased polyfunctional CD8+ T cell effector responses, and diminished ICT efficacy Schematic overview of the protocol used to assess the impact of antibiotic-induced gut microbiota depletion on ICT efficacy, gut microbiota translocation and effector CD8+ T cell response. C57BL/6 mice (female, 6–8 wks, Jackson) were treated ± antibiotics (ABX, 2 mg/ml streptomycin and 1500 U/ml penicillin G in drinking water) for 7d before B16-F10 tumor inoculation. Mice were treated with 200μg anti-PD-1 and 200μg anti-CTLA-4 mAb intraperitoneally on days 4, 8, and 12 after tumor implantation (**A**) Tumor volume of mice ± ABX and ICT. n=5–7 mice per group. (**B**) Bacterial load of MLN in mice + ABX and ICT, as determined by bacterial group (Eubacteria, all bacteria) quantitative-PCR of MLN gDNA. n=8 per group. Dotted line represents the limit of detection. Three-dimensional t-distributed stochastic neighbor embedding (t-SNE) plot of secretory cytokine profiles of CD8+ T cells isolated from (**C**) MLN (n=3 per group) and (**D**) TDLN (n=10 per group) of mice ± ABX + ICT, as determined by using Isoplexis IsoSpark; 28-plex mouse adaptive immune panel. Only CD8+ T cells secreting at least one cytokine are represented. tSNE plot displaying immune functional categories of CD8+ T cells from (**E**) MLN and (**F**) TDLN, defined as Effector: Granzyme B, IFN-γ, MIP-1α, TNF-α Stimulatory: GM-CSF, IL-12p70, IL-15, IL-18, IL-2, IL-21, IL-5, IL-7 Chemoattractive: BCA-1, CCL-11, IP-10, RANTES, CXCL1, CXCL13 Regulatory: FAS, IL-10, IL-13, IL-27, IL-4, sCD137 Inflammatory: IL-17A, IL-1β, IL-6, MCP-1 Absolute quantification of IFN-γ and Granzyme B secreting CD8+ T cells isolated from (**G**) MLN and (**H**) TDLN of mice ± ABX + ICT. For (**B**), each point represents individual animal. For (**C, D**, **E, F**), each point represents individual CD8+ T cells isolated from MLN and TDLN of mice ± ABX + ICT. Bars represent the mean ± SEM. Statistical analysis by Mann-Whitney test. *P<0.05, **P<0.01, ***P<0.001, ****P<0.0001.

We treated mice with or without antibiotics one week before tumor implantation and then proceeded with ICT (fig. 6A). Consistent with prior reports (12–14), antibiotic treatment induced an ICT hyporesponsive state in our preclinical cancer model (fig. 6A). Further, antibiotic exposure significantly reduced gut microbiota translocation into MLNs (p=0.0006, fig. 6B). To further investigate the immunological impact of antibiotic-induced gut microbiota depletion, we isolated CD8+ T cells from MLNs and TDLNs of control or antibiotic-treated mice and performed single-cell multiplex cytokine profiling (Isoplexis IsoSpark; 28-plex mouse adaptive immune IsoCode chip panel). Overall, the MLN and TDLN CD8+ T cell cytokine secretion profile was markedly distinct between the antibiotic-treated mice versus untreated controls (fig. 6C and D). Specifically, the number of CD8+ effector T cells (defined as T cells secreting IFN-γ, granzyme-B, MIP-1α (CCL3) and/or TNF-〈) was markedly reduced in antibiotic-treated mice (fig. 6E–F, fig. S20–21). Interestingly, T cell polyfunctionality, the ability of T cells to secrete two or more cytokines per cell, has been associated with positive clinical response to cancer immunotherapies (including ICT and CAR-T therapy) in mice and humans (58–61). Indeed, MLN and TDLN CD8+ T cells recovered from mice without antibiotic exposure and treated with ICT exhibited higher T cell polyfunctionality (fig. S22). Accordingly, the number of MLN and TDLN CD8+ T cells secreting interferon-γ (IFN-γ) or granzyme-B (GZMB) was lower in antibiotic-treated mice compared to untreated controls (fig. 6G and H).

We also measured the cytokine profiles of TDLN DCs. TDLN DCs isolated from antibiotic-treated mice secreted markedly lower levels of GM-CSF, IL-1β, IL-4, and IL-6 upon PMA and Ionomycin stimulation compared to TDLN DCs recovered from untreated controls (fig. S23). Of note, secretion of IL-12, a cytokine crucial for Th1 T cell differentiation and anti-cancer immunity, was also higher in TDLN DCs from untreated controls (~7 fold higher in signal intensity, p=0.073, fig. S23). These results suggest that antibiotic-induced depletion of endogenous gut bacteria and a subsequent decrease in bacterial translocation results in reduced ICT efficacy via altering DC activation and CD8+ T cell effector responses in secondary lymphoid organs.

Finally, we sought to determine whether the antibiotic-induced ICT-hyporesponsiveness observed above could be reversed by administering probiotic therapy with translocator species *E. faecalis* and *E. coli* versus a control probiotic *L. acidophilus* (fig. S24). Treatment with *L. acidophilus* mitigated ICT efficacy, resulting in no significant difference in tumor volume when compared to isotype treated controls (fig. S24A). In contrast, mice treated with translocators *E. coli* and *E. faecalis* showed improved ICT efficacy (as measured by tumor volume) when compared to mice treated with isotype control (fig. S24A), and smaller (albeit not significant) tumor volumes when compared to ICT alone (fig. S24A). Furthermore, mice treated with translocator species exhibited higher bacterial load within the tumor (fig. S24B). Of note, in the *E. coli*/*E. faecalis* translocator treated group, the majority of cultured tumor bacteria were identified as *E. faecalis*, further highlighting the translocation capacity of *E. faecalis* in this model.

## DISCUSSION

In this study, we have identified gut bacteria translocation into secondary lymphoid organs as a general mechanism by which resident bacteria in the gut can shape and dictate extra-intestinal anti-tumor immune responses in the setting of ICT. ICT enhances the ability of DCs to facilitate gut bacteria translocation into secondary lymphoid organs and also induces MLN remodeling. And specific gut bacteria taxa have a greater predilection or capacity to translocate, with a notable differential ability of some taxa to induce anti-cancer immune responses (fig. S25).

Numerous mechanisms are being posited by which gut bacteria can enhance anti-tumor responses in the setting of ICT: gut bacteria expressing peptide sequences homologous to tumor antigens/neoantigens (molecular mimicry) (23); gut bacteria-derived metabolites (SCFA (3), c-di-AMP (20), and inosine (9)); or direct activation of innate/adaptive immune cells to drive anti-tumor responses (22, 62). All of the aforementioned mechanisms require gut bacteria or gut bacteria-derived product engagement with host innate and/or adaptive immune effectors. The emerging concept of the tumor microbiome (63, 64) provides one possible explanation: resident bacteria or bacteria-derived metabolites within the tumor inducing anti-tumor effects. Of note, the relative abundance of Bacteroides (10–15%) and Firmicutes (40–70%) in our murine melanoma tumors are comparable to those observed in tumor microbiomes in human melanoma patients, 15% and 40% respectively (63). In addition, the highly abundant translocator taxa that we identified in our study, (Enterococcaceae, Lactobacillaceae, and Enterobacteriaceae) were also detected in melanoma tumors in humans (63). More intriguingly, these taxa identified in both murine and human specimens originate from the gut, as opposed to from adjacent tissues such as the skin. But an unanswered question is how do gut bacteria end up in such distal sites as the skin or lung?

One potential explanation is gut microbiota translocation. The ability of gut bacteria to translocate into secondary lymphoid organs, particularly MLN, has been well-described in infectious (65–68) and inflammatory diseases (69–71). Further, a seminal prior study highlighted the importance of gut bacteria translocation into secondary lymphoid organs for optimal cyclophosphamide immune-mediated anti-cancer responses, identifying a novel mechanism by which this commonly used alkylating chemotherapeutic agent induces host immune anti-cancer effects (24, 72). Our data suggest that an analogous process may be one mechanism by which ICT promote gut bacteria translocation into secondary lymphoid organs: ICT-induced gut inflammation creating an environment in which gut bacteria are more readily able to translocate into secondary lymphoid organs to engage innate and adaptive immune effectors.

Interestingly, while bacteremia, and the development of sepsis, is generally associated with increased morbidity and mortality, it has been associated with improved cancer outcomes in both preclinical models (73) and in patients (74, 75). And localized infections, proximal to primary tumors, have also been associated with improved clinical outcomes in animals (dogs with osteosarcoma (76)) and humans (head and neck cancer (77) and osteosarcoma (78)). Yet the development of bacteremia has not been associated with ICT treatment. And our data did not identify any appreciable deficit in gut barrier integrity with ICT. In contrast, a previous study reported a reduction in transepithelial electrical resistance after anti-CTLA-4 treatment, suggesting reduced intestinal barrier integrity with anti-CTLA-4 treatment (9). Nonetheless, in addition to gut bacteria translocation into secondary lymphoid organs, a low-grade and subclinical gut bacteria translocation into the systemic circulation (as suggested in fig. 5E) could be an additional potential mechanism by which tumor microbiomes develop.

While the role of DCs in anti-cancer immunity is well-established, our data provide an additional layer of insight in that gut bacteria-mediated anti-cancer immune effects may also be dependent on DCs capacity to carry or traffic gut bacteria. Previous studies have reported that gut inflammation increases DC mobilization into MLNs and TDLNs in a CCR7 dependent manner (39, 40). Our data corroborate these findings and also add that the degree of gut bacteria translocation (bacterial burden) into secondary lymphoid organs, especially MLN, is DC dependent. Additionally, a recent study reported the frequent intracellular localization of microbes in cancer and immune cells (63), thus further supporting the role of DCs in bacterial translocation.

MLN serves as an “intestinal mucosal firewall,” restricting gut-derived antigens within the MLN (36, 54). Interestingly, tumor development and inflammation (49–52) lead to distinct remodeling of lymph node architecture characterized by lymphangiogenesis and HEV dilation/dedifferentiation. Here, we report a similar MLN remodeling process which is induced by ICT. A potential mechanism for this process is that ICT induces DC recruitment into MLN (fig 4F), and lymph node endothelial expansion is induced by DCs (53). It was, however, surprising that gut bacteria traffic to the MLN appears to be DC dependent, but translocation from MLN and beyond was not. These results raise an intriguing possibility of whether ICT-induced MLN remodeling may result in a breach of the mucosal firewall and lead to increased dissemination of bacteria from MLN to extraintestinal sites, including the TDLN and tumor, via hematogenous spread. Thus, ICT may also provide a previously unappreciated innate immune activation effect, lymph node remodeling, leading to increased bacterial translocation into TDLN and tumors, in addition to the well-studied T cell specific effects.

A limitation of this study is that these phenomena were observed in a melanoma preclinical model, and it is unclear whether our findings are generalizable to other tumor models. Further, while combination ICT (anti-PD-1 and anti-CTLA-4) has been shown to be more effective than single agent ICT in melanoma (1, 2), combination therapy is also associated with a higher incidence of immune-related adverse events, including colitis, in cancer patients (79). Thus, the magnitude of gut microbiota translocation observed with single agent ICT could be quite different.

In summary, by illuminating a general mechanism by which gut bacteria shape or influence extraintestinal anti-cancer immune responses, our results could provide insight as to why different gut microbiota taxa or mechanisms are being espoused as critical for ICT efficacy. Ultimately, multiple gut bacteria taxa and/or mechanisms have the potential to drive host anti-cancer immune responses, but one common prerequisite is that these microbes or microbe-derived metabolites must engage with key components of the innate and adaptive immune systems. As such, ICT and DC-mediated gut bacteria translocation into secondary lymphoid organs could serve as a fundamental mechanism by which these processes transpire. The general insights gained by this study are unlikely to be specific a particular immunotherapy or tumor. In the future, these principles could also apply to understanding if and how gut microbiota modulate the efficacy of other cancer immunotherapies.

## MATERIALS AND METHODS

### Study design

The goal of this study was to evaluate if and how gut bacteria translocation into secondary lymphoid organs modulates ICT efficacy in a preclinical melanoma model. Mice of different genotypes were used for tumor response experiments, with approved humane end points to terminate *in vivo* experiments, and for harvesting of mouse tissue (tumor and immune cells) for histopathology and *ex vivo* immune functional profiling assays. The number of samples combined and the number of independent experiments are included in the figure legends.

#### Mice.

C57BL/6J (Stock No: 000664), B6.FVB-1700016L21RikTg (Itgax-DTR/EGFP)57Lan/J) (CD11c-DTR, Stock No: 004509) (37), C57BL/6-Tg(TcraTcrb)1100Mjb/J (OT-1, Stock No: 003831) (80), and B6.129P2(C)-Ccr7tm1Rfor/J (CCR7−/−, Stock No: 006621) (81) mice were obtained from Jackson Laboratories and bred and maintained in the barrier facility at the University of Texas Southwestern Medical Center. All animals were kept on a 12-hour light-dark cycle and were fed standard mouse chow (Teklad 2916, irradiated). Sex-matched, 6–8 week old mice were used for all experiments and co-housed littermates were used as controls. The resource equation method was used for sample size determination (82, 83). Experiments were performed using protocols (APN 2017–102122) approved by the Institutional Animal Care and Use Committees of the UT Southwestern Medical Center.

#### Preclinical model of melanoma and immune checkpoint therapy.

B16-F10 cells (ATCC CRL-6475; RRID:CVC_0159) were grown at 37°C under 5% CO2 in DMEM medium supplemented with 10% heat-inactivated FBS (Sigma), 100 units/ml penicillin, 100μg/ml streptomycin sulfate, 2mM L-glutamine, 1mM sodium pyruvate. C57BL/6J mice were fed sterile or antibiotic-supplemented (2 mg/ml streptomycin and 1500 U/ml penicillin G, Sigma) water. 1 ×10^5^ B16-F10 cells were implanted subcutaneously into the right flank of mice. Four days after the tumor inoculation, mice were injected with 200μg anti-PD-1 antibody (RMP1–14, CD270, BioXcell) and anti-CTLA-4 antibody (9D9, CD152, BioXcell) or isotype control (Rat IgG2a, κ or Mouse IgG2b, respectively) intraperitoneally; an additional two treatments were given with 4d intervals. Tumor volumes were calculated using measurements from a digital caliper and the following formula: π/6 × length × width × height (84). Loss of survival was defined as death (with moribund mice being euthanized) or when tumor diameter > 2 cm in any dimension.

### Gut and Tissue Microbiome Profiling

#### Cultured Microbiota Enumeration.

Mice were euthanized and tissue samples (MLN, TDLN, spleen, and tumor) were resected and weighed (2 mL screw-cap microtube with 500ul sterile reduced PBS and one sterile 5mm borosilicate glass beads (Sigma)). Samples were kept in a 2.5L portable anaerobic chamber with anaerobic gas packs (Mitsubishi Gas Chemical) during sample collection and transportation. Tissue samples were homogenized using TissueLyser II (Qiagen) at 30 Hz for 3 minutes; serially diluted in reduced PBS; and plated and incubated on BHI/Blood, YCFA, and CME0151 agar for 24–72 hours at 37°C under anaerobic conditions. Colony-forming units (CFUs) were counted.

#### 16S rRNA sequencing and analysis.

gDNA was extracted from fecal and tissue samples using the MagAttract Power Microbiome DNA/RNA KF kit (Qiagen) and Kingfisher Flex (Thermo Fisher Scientific). 16S rRNA genes (variable region 4, V4) were amplified from each sample, sequenced, and analyzed as previously described (85). Please see Supplementary Materials for more details.

#### Quantitative PCR for tissue microbiome analysis.

Bacterial load in tissue was quantified by qPCR analysis (SsoAdvanced SYBR Green Supermix, Bio-Rad) of microbial gDNA using the universal 16S rRNA gene as previously described (85). Please see Supplementary Materials for more details.

#### Surgical removal of secondary lymphoid organs.

1 × 10^5^ B16-F10 cells were implanted subcutaneously into the right flank of mice. On day 7 post tumor implantation, mice with tumor volumes of 100 mm^3^ ± 20 mm^3^ were randomized to 1) mesenteric lymph nodes (MLN) resection; 2) spleen resection; 3) inguinal lymph node (TDLN), defined as the right inguinal lymph nodes; or 4) sham, longitudinal abdominal incision only. Mice were anesthetized with vaporized isoflurane. Abdominal fur was removed using an electric razor followed by hair removal cream (Veet). The abdomen was then cleaned with betadine and 70% ethanol. Mice were covered with sterile surgical drapes, and a longitudinal abdominal incision was then performed. Intestines were gently removed from the peritoneal cavity and placed on moistened sterile gauze pads (with sterile normal saline). MLN, TDLN, or spleen was carefully resected with surgical scissors and/or a cauterizer (Medline). Large blood vessels were cauterized and ligated. The abdominal wall was closed using absorbable sutures (5/0 PGA, Covetrus) in individual stitches; skin closed using nonabsorbable sutures (5/0 Monofilament nylon, Covetrus) and tissue adhesive (veterinary surgical adhesive, Covetrus). The incision was then coated with triple antibiotic ointment. Aanalgesic carprofen (5 mg/kg) was administered intraperitoneally immediately after surgery. Anesthesia was discontinued. Mice were initially placed on a heated surgical bed for recovery, but once moving transitioned to a heated cage. Mice were monitored closely and evaluated for pain and lethargy. Any moribund mouse (e.g. signs of lethargy, cool to touch, etc.) was immediately euthanized. At a 4-hour post-surgery check, buprenorphine (0.05 mg/kg SQ) was administered as needed for pain. For the first 72 hours, mice were monitored twice daily for pain management and infection monitoring. Mice found dead or euthanized for being moribund within the first 72 hours after surgery were not included in the final analysis, as mortality was attributed to either post-surgical bleeding or infection.

#### Intra-MLN injection of GFP expressing *Escherichia coli*.

*Escherichia coli* expressing GFP (harboring pZe21-RBSmod + sfGFP) were grown in tryptic-soy broth with 50 ug/mL kanamycin aerobically at 37°C for 24 hours. Bacterial cells were harvested (2000 × G, 4°C) and washed three times with ice-cold sterile PBS. 1×10^5^ B16-F10 cells were implanted subcutaneously into the right flank of C57BL/6J mice. On day 7 post tumor implantation, mice with tumor volumes of 100 mm^3^ ± 20 mm^3^ were then randomized. 3 doses of 200μg anti-PD-1 antibody (RMP1–14, CD270, BioXcell) and anti-CTLA-4 antibody (9D9, CD152, BioXcell) were given i.p. and daily for three consecutive days. GFP+ *E. coli* injection was performed 1 day after the last dose of ICT. Mice underwent abdominal surgery as described above. 1 × 10^7^ GFP expressing *E. coli* were injected directly into the one or two nodes of MLN. After 24 hours post injection, mice were sacrificed and tumor, TDLN and blood were collected. Tissue homogenates and blood were spread on tryptic-soy agar plate with 50 ug/mL kanamycin and cultured aerobically for 24–48 hours at 37°C. GFP expressing colonies were enumerated.

#### Single cell suspension preparation from secondary lymphoid organs.

Dissected MLN and TDLN were stored on ice-cold sterile PBS. Tissue samples were mashed (with the plunger of a 10 ml syringe) and filtered (70 μm sterile cell strainer, Fisher) over a sterile petri dish. Cell strainer and plunger were washed with additional 15 ml of ice-cold PBS. The cell suspension was then collected from the petri dish and transferred to 15ml conical tube. The cell pellet was harvested (300 G, 4°C for 10 min) and resuspended in ice-cold PBS.

#### Tumor infiltrating lymphocytes isolation.

Single cells from dissected tumor tissue were isolated using the Miltenyi Biotec gentleMACS^™^ Dissociators. Briefly, the tumor was cut into 2–4 mm pieces; mechanically dissociated; and then enzymatically digested using tumor dissociation kit enzyme D, R and A at 37°C for 40 min. Tumor tissue was further mechanically dissociated and filtered (70 μm sterile cell strainer). The single-cell suspension (10 ml of RPMI) was then centrifuged at 300 G, 4°C for 10 min. The cell pellet was suspended in RBC lysis buffer (Invitrogen) and incubated at RT for 2 min to remove erythrocytes. Cells were washed (10ml of PBS) and centrifuged at 300 G, 4°C for 10 min. Cell pellets were suspended in 40% Percoll solution and carefully transferred to 15ml conical tubes containing 80% Percoll solution. Suspensions were centrifuged at 300 G at RT for 20 min (ascending rate: 5; descending rate: 0). Cells at the interface between 40% and 80% Percoll solutions were carefully collected and washed once with PBS.

#### Flow cytometry analysis.

Single-cell suspensions of cells were transferred to U-bottom 96 well plate (Corning) and centrifuged at 300 G, 4°C for 10 min. For surface staining, cells were incubated with zombie-yellow live/dead dye (Biolegend) diluted in PBS (1:500) at RT for 15 minutes. After washing with PBS, cells were incubated with Fcγ receptor blocking antibody (clone 2.4G2, BD Biosciences), diluted in FACS buffer, PBS supplemented with 2% heat-inactivated fetal bovine serum (FBS) and 2mM EDTA, for 10 min at 4°C in the dark followed by surface staining for 30 min at 4°C in the dark. Cells were then washed with FACS buffer twice and suspended in FACS buffer for flow cytometry. For intracellular staining, cells were incubated in 200μl RPMI supplemented with 10% FBS, 50 μM 2-mercaptoethanol, 50ng/ml Phorbol 12-Myristate 13-Acetate (PMA), 750ng/ml Ionomycin and 10μg/ml Brefeldin-A (all Sigma) at 37°C with 5% CO2 for 4 hours. After the incubation, cells were washed with PBS. Cells were then incubated with zombie-yellow live/dead dye (Biolegend) diluted in PBS (1:500) at RT for 15 minutes. After washing with PBS, cells were incubated with Fcγ receptor blocking antibody (clone 2.4G2, BD Biosciences), diluted in FACS buffer for 10 min at 4°C in the dark followed by surface staining for 30 min at 4°C in the dark. Cells were then fixed and permeabilized using the eBioscience^™^ Foxp3 Fixation/Permeabilization kit (eBioscience) according to the manufacturer’s protocol. Cells were then stained with antibodies targeting intracellular proteins for 30 min at RT. Prior to the acquisition, cells were washed with FACS buffer and flow cytometry was performed on a BD FACSLyric. Flow cytometry gating strategy for the various experiments used in this study are detailed in fig. S11, S12, and S14. Antibodies used for flow cytometry are listed in Table S2.

#### Expansion of mouse splenic DCs by B16-FLT3L implant.

For the generation of spleen-derived dendritic cells (DCs), 5 × 10^6^ B16-FLT3L cells (RRID:CVCL_IJ12) were injected subcutaneously into the right flank of C57BL/6J mice. After 10–16 d, spleens were harvested. CD11c+ DCs were isolated using CD11c microbeads and magnetic-activated cell sorting (Miltenyi). Of note, B16-FLT3L injection did not induce CD11c+ DC maturation, as reported previously (28, 86), as evidenced by a lack of proliferation of non-stimulated CD11c+DCs.

#### *Ex vivo* immune cell priming assay.

For the DC stimulation assays, CD11c+ DCs were stimulated with different bacterial lysates for 6 hours at 37°C under 5% CO2 and then washed with PBS. The surface expression of DC activation markers was measured by flow cytometry as described above. For T cell priming assay, naïve CD8+ T cells were isolated from the spleens of OT-I (C57BL/6-Tg(TcraTcrb)1100Mjb/J) mice (female, 6–8 weeks age). Cells were maintained in complete RPMI-1640 medium (Sigma) supplemented with 10% FBS, 55μm 2-Mercaptoethanol (Sigma), 100 units/ml penicillin and 100 μg/ml streptomycin sulfate at 37°C with 5% CO2. CD11c+ DCs were pulsed with different bacterial lysates and OVA 257–264 peptide for 6h followed by thorough washing, and then co-cultured with naïve OVA 257–264 specific OT-I CD8+ T cells for 7 days. After co-culture, cells were washed with PBS. T cell activation and IFN-γ production were measured by flow cytometry as described above.

#### Single-cell multiplex cytokine profiling of murine T cells.

Lymphocytes from MLNs and TDLNs of mice (C57BL/6J, female, 6–8 weeks old) treated ± antibiotics in the drinking water (2 mg/ml streptomycin and 1500 U/ml penicillin G) and implanted with B16-F10 tumors and treated with ICT (anti-PD1 and anti-CTLA-4 antibody treatment) were collected at one day after the last ICT and pooled into one sample per experimental group. Pooled lymphocytes were then cultured overnight in recombinant murine-IL-2 (1μg/ml, Peptrotech)-supplemented RPMI 1640 medium. CD8+ T cells were then isolated using CD8 microbeads (Miltenyi) and stimulated with immobilized anti-mouse CD3 (Invitrogen) and soluble anti-mouse CD28 (Invitrogen) at 37 °C, 5% CO2 for 48 h. Approximately 30,000 CD8+ T cells were loaded onto an IsoCode chip (IsoPlexis, New Heaven, CN) containing ~12,000 microchambers pre-patterned with a 28-plex antibody array, imaged for single cell location in microchambers and incubated at 37 °C, 5% CO2 for additional 16 h. Following the incubation period, ELISA detection was used to determine which combinations of proteins were being secreted by each individual cell. Secreted proteins from single cells were captured by antibody-barcoded slides; the polyfunctional profile (2+ proteins per cell) of single cells was evaluated by IsoPlexis’ software.

#### *In vivo* Dendritic Cell Depletion.

For dendritic cell depletion experiment, CD11c-DTR mice were intraperitoneally injected with 100ng of diphtheria toxin (Sigma) as previously described (37). For confirming the dendritic cell depletion, spleens from the wild-type mice and CD11c-DTR mice injected with diphtheria toxin or PBS were resected 48 hours after diphtheria toxin injection and CD11c expression among the splenic lymphocytes were determined by flow cytometry.

#### Histology.

C57BL/6J mice were implanted with subcutaneous B16-F10 tumors and treated with 3 doses of ICT (200 ug anti-PD-1 and 200 ug anti-CTLA-4) or IgG Isotype control. 24 hours after the third dose of ICT, mice were euthanized and paired MLNs and TDLNs from individual mice were collected and placed into individual histology cassettes. Histology cassettes were immediately immersed in 4% paraformaldehyde in PBS and placed on a laboratory rocker at RT for 48 hours. Samples were transferred to 70% ethanol. The fixed lymphoid tissue was dehydrated, cleared, and infiltrated with paraffin. Samples were embedded for maximum surface area and sectioned longitudinally to optimize visualization of lymphatics running parallel to the plane of section. 5μm serial sections were collected for routine hematoxylin and eosin (H&E) and MECA-79 immunohistochemistry. H&E regressive staining was performed using Leica-Surgipath Selectech reagents (Hematoxylin 560, Define Concentrate, Blue Buffer, Alcoholic Eosin Y 515, Deer Park, IL) on a Sakura DRS601 x-y-z robotic stainer.

#### Immunohistochemistry (IHC).

To identify high endothelial venules (HEVs) within lymph nodes, serial sections from the same tissue block used for H&E slides were obtained for IHC for the HEV-specific marker MECA-79. In detail, slides were deparaffinized in xylene and brought to water in graded ethanols prior to heat-mediated antigen-retrieval. MECA-79 antigenic epitopes in the lymph-node sections were revealed by 25-minute exposure to steam heated pH 6.0 antigen retrieval citra (Biogenex). After cooling, sections were buffered in PBS, and endogenous peroxidases quenched in 0.3% H_2_O_2_. Slides were washed with PBS and blocked against non-specific secondary binding with 2.5% normal goat serum for 30 minutes. Sections were then incubated with rat anti-MECA-79 (SC-19602, Santa Cruz) diluted 1:500 in PBS overnight at 4°C. Following overnight incubation, slides were washed in PBS and bound MECA-79 primary was detected with Peroxidase Polymer conjugated Goat anti-Rat IgG (MP-7444–15, Vector Laboratories) for 10 minutes and diaminobenzidine chromagen (SK-4103, Vector Laboratories) for 5 minutes with interceding PBS washes. MECA-79 stained sections were counterstained with light hematoxylin, dehydrated, cleared, and coverslips affixed with synthetic mounting media.

#### Light Microscopy and Image Quantification.

Both H&E and MECA-79 IHC slides were imaged using an Aperio CS2 slide scanner (Leica Biosystems) at 40x magnification. Images were obtained at 10x and 20x using ImageScope software version 12.3 (Leica Biosystems). To confirm differences in HEV dilation/dedifferentiation and lymphangiogenesis, images were independently reviewed by pathologist Bret M. Evers M.D. Ph.D., UTSW Histopathology Core, who was blinded to the treatment groups. For HEV diameter quantification, the longest axis of individual MECA-79 positive HEVs were measured using the ImageScope measurement tool. For quantification of differences in lymphangiogenesis, the total number of blood vessels per MLN were counted on the H&E images.

#### Statistical analysis.

GraphPad Prism v.9.2 was used for statistical analysis. All data sets were tested for normality (e.g. Shapiro-Wilk). Data sets with normal distribution were analyzed with parametric tests, such as standard student t-test or one-way ANOVA with Bonferroni post-test. For non-normal distributions, non-parametric tests, such as Mann Whitney U test or Kruskal Wallis with Dunn’s post-test, were applied. Two-way ANOVA with Bonferroni post-test was used for tumor growth curves. Survival was analyzed using the Mantel-Cox Log-rank test.

## Supplementary Material

data file S1

reproducibility checklist

main supplementary materials

## Data Availability

The 16S rRNA sequencing data for this study has been deposited in the NCBI Sequence Read Archive: http://www.ncbi.nlm.nih.gov/sra/PRJNA886750, http://www.ncbi.nlm.nih.gov/sra/ PRJNA886832. Requests for bacterial strains should be addressed to A.Y.K and should be covered by a material transfer agreement. All genetically engineered mice used in this study are commercially available. All data needed to validate the conclusions in this study can be found in the paper or Supplementary Materials.
