# Supplementary material for: Immune checkpoint blockade induces gut microbiota translocation that augments extraintestinal anti-tumor immunity
Source: Sci Immunol. Author manuscript; Available in PMC 2023 Apr 7. (PMC10080670; doi:10.1126/sciimmunol.abo2003)
Supplement: main supplementary materials [file NIHMS1887590-supplement-main_supplementary_materials.pdf]

Supplementary Materials for

Immune checkpoint blockade induces gut microbiota translocation that augments extraintestinal anti-tumor immunity

Yongbin Choi, Jake N. Lichterman, Laura A. Coughlin, Nicole Poulides, Wenling Li, Priscilla Del Valle, Suzette N. Palmer, Shuheng Gan, Jiwoong Kim, Xiaowei Zhan, Yajing Gao, Bret M. Evers, Lora V. Hooper, Chandrashekhar Pasare and Andrew Y. Koh*

*Correspondence to: andrew.koh@utsouthwestern.edu

This PDF file includes:

Materials and Methods
Figures S1-S25
Table S1-S2

Supplementary Materials and Methods

16S rRNA Sequencing Library Preparation

We used the reverse primer 926R, 5'-*CAAGCAGAAGACGGCATACGAGAT*-NNNNNNNN-**AGTCAGTCAG-CC-GGACTACHVGGGTWTCTAAT**-3": the italicized sequence is the reverse MiSeq primer i7; NNNNNNNN designates the unique 8-base barcode used to tag each PCR product; the bold sequence is the broad-range 16S bacterial primer containing the pad-link-16SR. The forward primer used was 515F, 5'-*AATGATACGGCGACCACCGAGA TCTACAC*-NNNNNNNN-**TATGGTAATT-GT-GTGCCAGCMGCCGCGGTAA**-3': the italicized sequence is MiSeq Primer i5; the NNNNNNNN designates the unique 8-base barcode used to tag each PCR product; and the bold sequence is the broad range 16S bacterial primer containing the pad-link-16SF. PCR reactions consisted of 17ul Accuprime Pfx Supermix, 1000 nM of each primer, and 20ng of template. Reaction conditions were 2 min at 95°C, followed by 30 cycles of 20 s at 95°C, 15 s at 55°C, 5 min at 72°C, then 10 min at 72°C, and a hold at 4°C on an Eppendorf Mastercycler. For tissue and tumor microbiome profiling, two rounds of PCR amplification were performed. Products were verified on a 1% agarose gel, and normalized using the AmPure Normalization plate protocol using the KingFisher Flex platform. Each plate was then pooled into a single tube, and the PCR product size and library quality of each individual pooled plate was checked using Agilent Technologies D1000 ScreenTape electrophoresis. Additionally, KAPA Biosystems PCR Library Quantification kit was used to quantify each pooled plate. Illumina spike-in (PhiX) was included at 4 pM at 10%, and the pooled sample library was included at 4pM at 90% yielding a final library concentration of 3.6 pM and PhiX concentration of 0.4 pM.

Gastrointestinal barrier function assays.

FITC-dextran permeability assay. Mice were fasted overnight. FITC-dextran (500 mg/kg; Sigma Aldrich; 4 kD) was administered via oral gavage. Mice were kept without food and water for 4 hours. Blood samples were obtained by terminal cardiac puncture and collected in BD Vacutainer SST tube (BD) 4 hours after FITC-dextran administration. Blood samples were centrifuged at 2,000xG at RT for 10 min. Serum was collected by taking the upper layer after centrifugation. The serum fluorescence intensity was measured at an excitation wavelength of 485 nm and an emission wavelength of 528 nm using a spectrophotometer (Synergy HT, BioTek).

Quantitative real-time PCR (qPCR). Total RNA was isolated from MLN, TDLN and tumor of wild-type, CCR7-deficient, CD11c-DTR, MLN-resected mouse treated with or without ICT or antibiotics. cDNA was synthesized from purified RNA using iScript cDNA synthesis kit (Bio-Rad). qPCR was performed using SsoAdvanced Universal SYBR Green Supermix (Bio-Rad) on a CFX96 Real-Time System (Bio-Rad). Relative expression values were determined using the comparative Ct (ΔΔCt) method (*87*) and transcript abundances were normalized to 18S rRNA transcript abundance. Amplification of target genes was conducted using following primers. Mouse 18s rRNA forward: 5’-CATTCGAA- CGTCTGCCCTAT-3’, mouse 18s rRNA forward reverse: 5'-CCTGCTGCCTTCCTTGGA-3’ mouse ZO-1 forward: 5’-ACCCGAAACTGATGC-TGTGGATAG-3’, mouse ZO-1 reverse: 5’-AAATGGCCGGGCAGAACTTGTGTA-3’, mouse TNF-α forward: 5’-TCTCATGCACCACCATCAAGGACT-3’, mouse TNF-α reverse: 5’-TGA-CCACTCTCCCTTTGCAGAACT-3’, mouse IL-1β forward: 5’-AAGGGCTGCTTCCAAACCT-TTGAC-3’, and mouse IL-1β reverse: 5’-ATACTGCCTGCCTGAAGCTCTTGT-3’.

Reagents and Resources. A list of key reagents and resources used for this study are listed in Table S2.

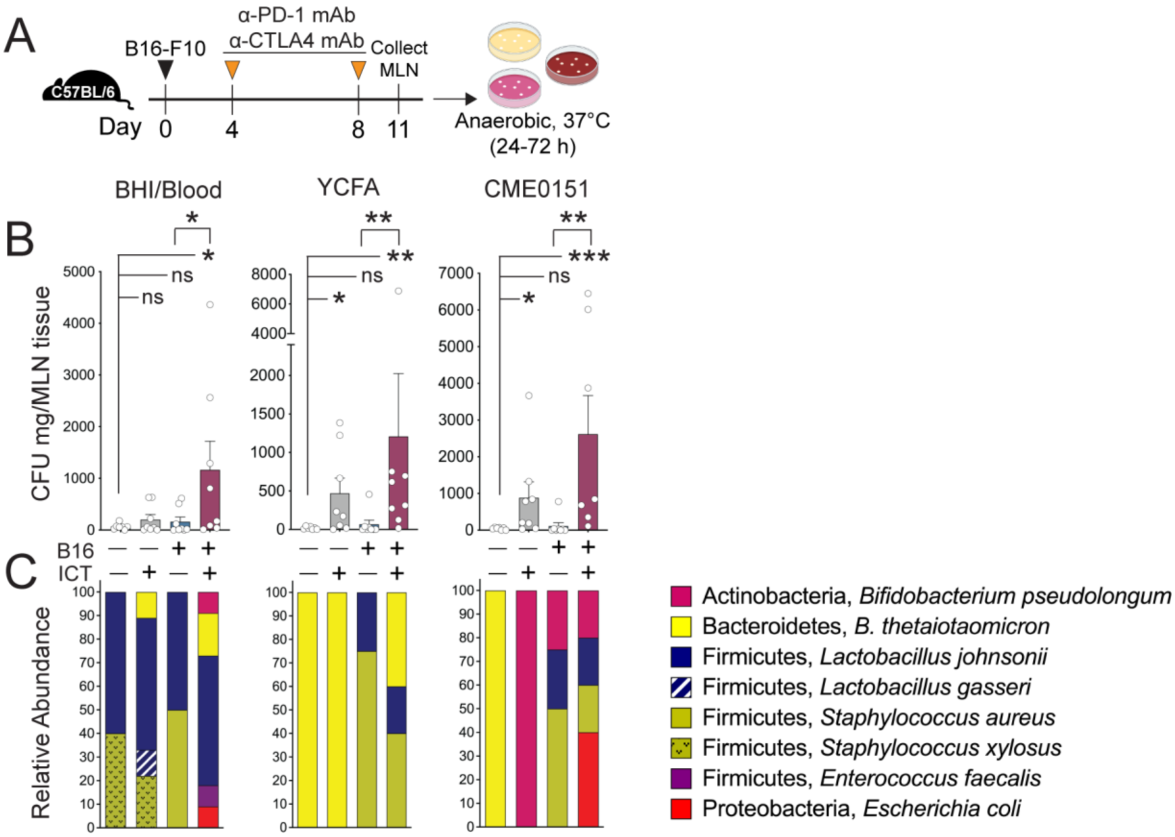

Figure S1. Immune checkpoint inhibitor therapy (ICT) induces bacterial translocation into mesenteric lymph nodes (MLN)

(A) Schematic overview of the protocol used to assess and quantify bacterial translocation into MLN in C57BL/6J mice (female, 6-8 wks, Jackson) bearing B16-F10 melanoma tumors and receiving ICT (200 ug anti-PD-1 and 200 ug anti-CTLA-4 mAb).

(B) Cultured bacterial levels in MLN. MLN tissue homogenates were serially diluted, plated on BHI/Blood, YCFA, and CME0151 agar media and incubated at 37℃ under anaerobic conditions for 24-72 hours. Quantification of colony-forming unit (CFU) from each agar plate was normalized to the tissue weight. n=7-8 per group. Points represent values from individual animals. Bars represent the mean ± SEM. Statistical analysis by Mann-Whitney test. *P<0.05, **P<0.01. ***, P<0.001.

(C) Relative abundance of cultured bacteria. Representative colonies (10-20 colonies with similar morphology and/or color) on each agar plate were selected and subsequently cultured in corresponding liquid media. gDNA was extracted. Full-length 16s rRNA gene (V1-V9 region) was amplified, purified, and sequenced (Sanger sequencing). Sequences were entered into the NCBI standard nucleotide Basic Local Alignment Search (BLAST) tool utilizing the rRNA/ITS databases. Bacterial species identification was ascertained from BLASTN results with the highest Total Score, with percent identity score >95% and E value <0.01.

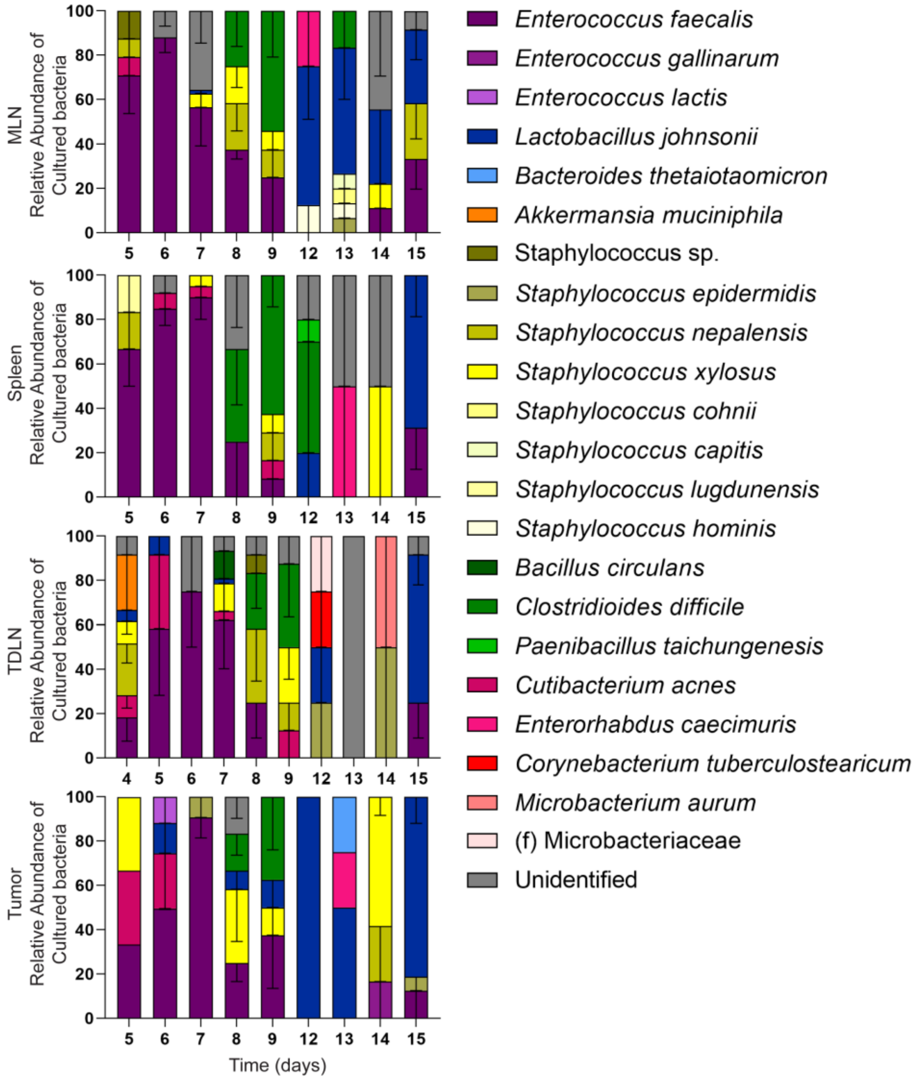

Figure S2. Tissue and tumor microbiomes (cultured microbiota) in mice bearing B16-F10 tumors and receiving ICT, as described in fig 1A-C.

Relative abundance of cultured bacteria from secondary lymphoid organs and tumor tissue recovered from C57BL/6J mice (n=6-8) bearing melanoma tumors and receiving anti-PD-1 and anti-CTLA-4 antibody treatment, as described in fig 1A. Tissue homogenates were serially diluted in reduced PBS and plated on YCFA agar and incubated for 24-72 hours at 37℃ under anaerobic conditions. Colony-forming units (CFUs) were counted. Representative colonies based on morphology (~10-20 for each morphology type) were picked and cultured in YCFA medium for additional 24-48 hours at 37℃ under anaerobic conditions. gDNA was isolated from bacterial cultures. Full-length 16s rRNA gene (V1-V9 region) was amplified, purified, and sequenced (Sanger sequencing). Sequences were entered into the NCBI standard nucleotide Basic Local Alignment Search (BLAST) tool utilizing the rRNA/ITS databases. Bacterial species identification was ascertained from BLASTN results with the highest Total Score, with percent identity score >95% and E value <0.01.

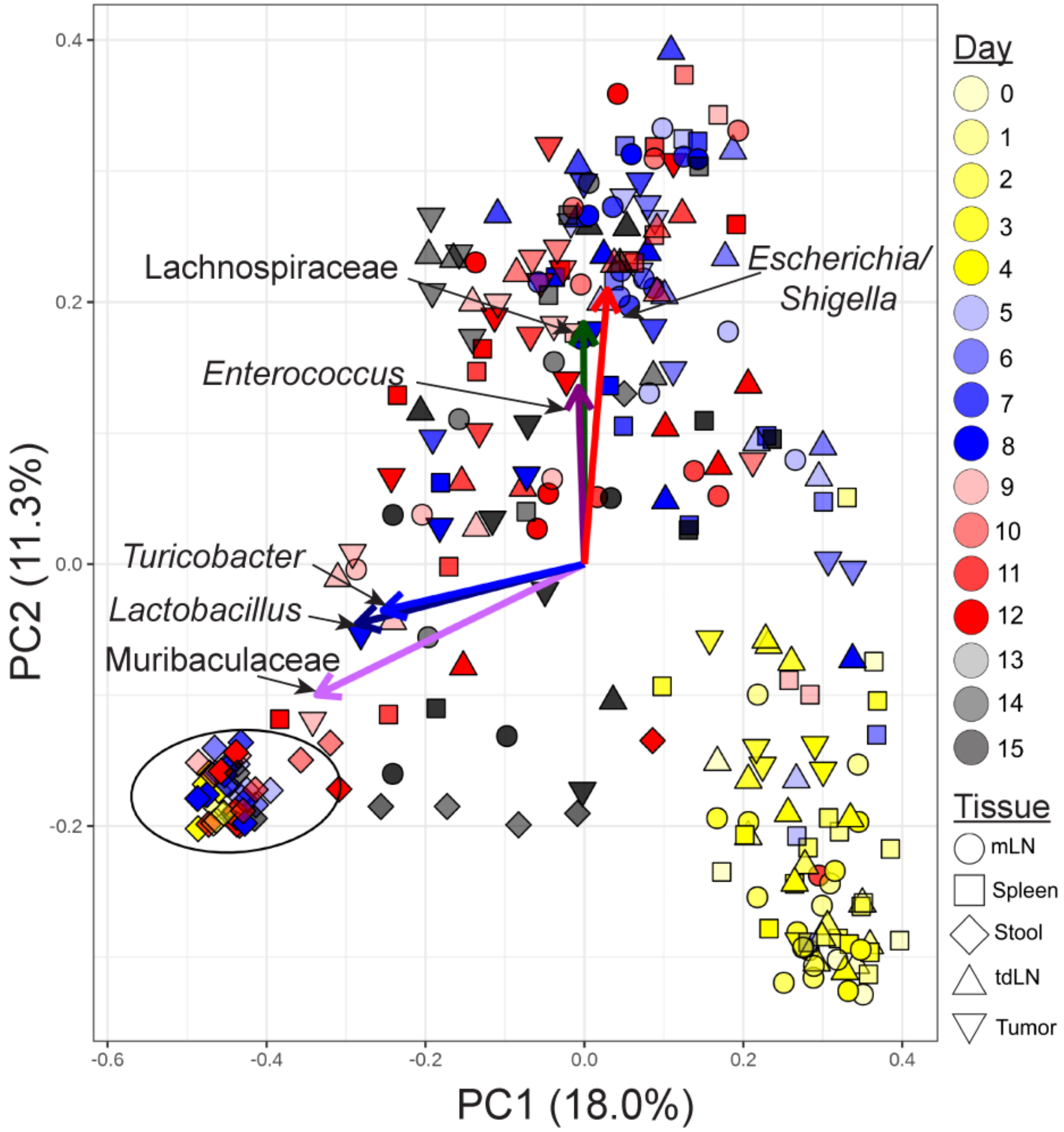

Figure S3. Principal coordinate analysis of tissue and gut microbiome of mice bearing melanoma tumor and receiving ICT

Principal coordinate analysis of tissue and gut 16S rRNA sequencing data (as detailed in fig. 1), weighted and normalized by Bray-Curtis distances. The proportion of variance accounted by each principal component is indicated. Vector analysis (as indicated by arrows) performed by singular value decomposition of 16S rRNA sequencing data interpreted visually as a linear biplot.

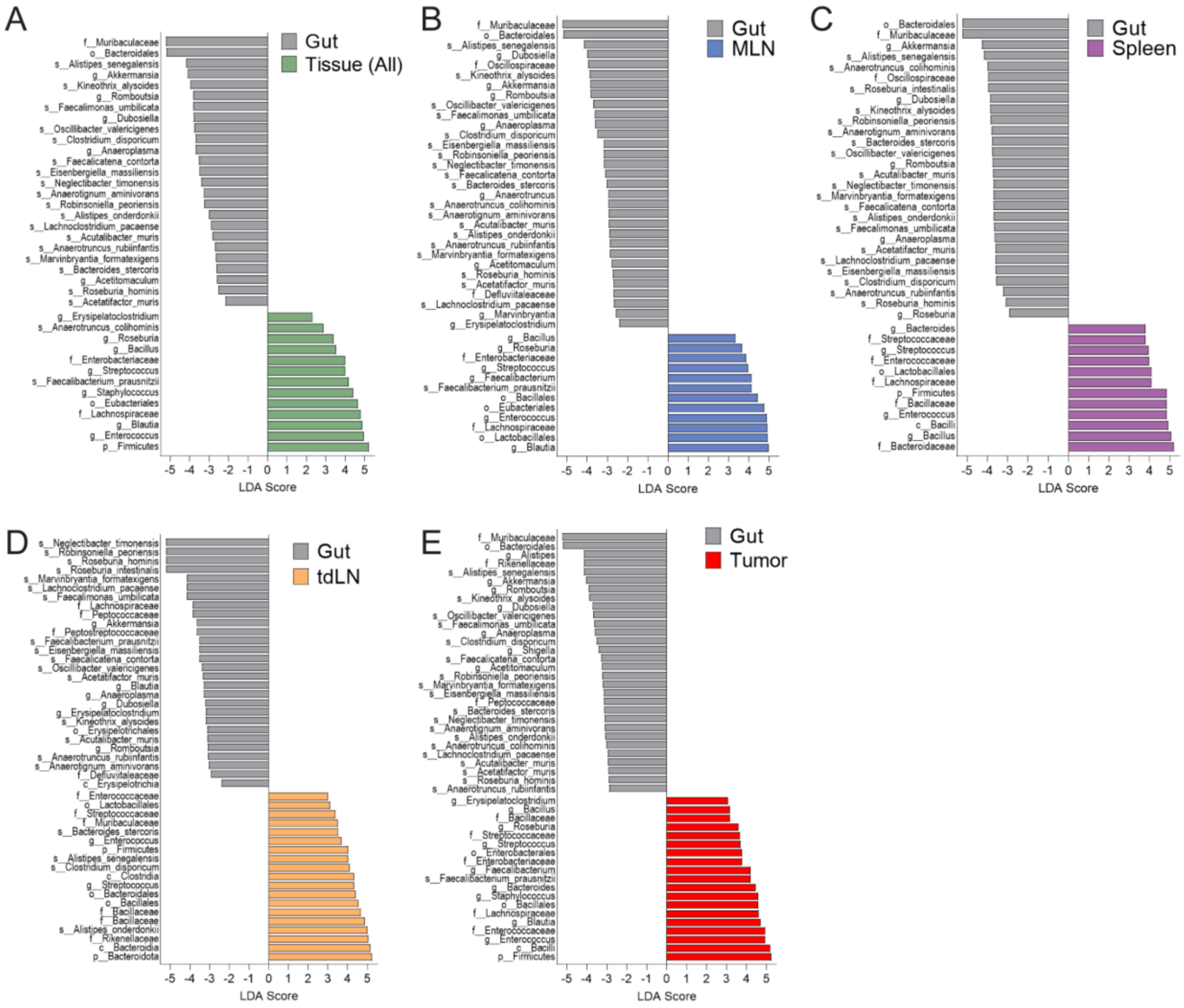

Figure S4. Comparison of gut and tissue microbiomes from mice bearing melanoma tumors and receiving anti-PD-1 and anti-CTLA-4 antibody treatment.

Microbiome composition determined by analysis of 16S rRNA sequencing (V4 region) of tissue and fecal samples recovered from C57BL/6J mice bearing melanoma tumors and receiving anti-PD-1 and anti-CTLA-4 antibody treatment, as in fig 1D-E. Differential bacterial taxonomic abundance between tissues types was analyzed by linear discriminant analysis effect size (LEfSe) projected as histograms. All listed bacterial groups (class (c), order (o), family (f), genus (g), or species(s)) were significantly enriched (>2 log-fold increase in linear discriminate analysis, LDA, score and $P<0.05$, Kruskal-Wallis test).

(A) Gut versus all other tissue types

(B) Gut versus mesenteric lymph node (MLN)

(C) Gut versus spleen

(D) Gut versus tumor-draining lymph node (TDLN)

(E) Gut versus tumor

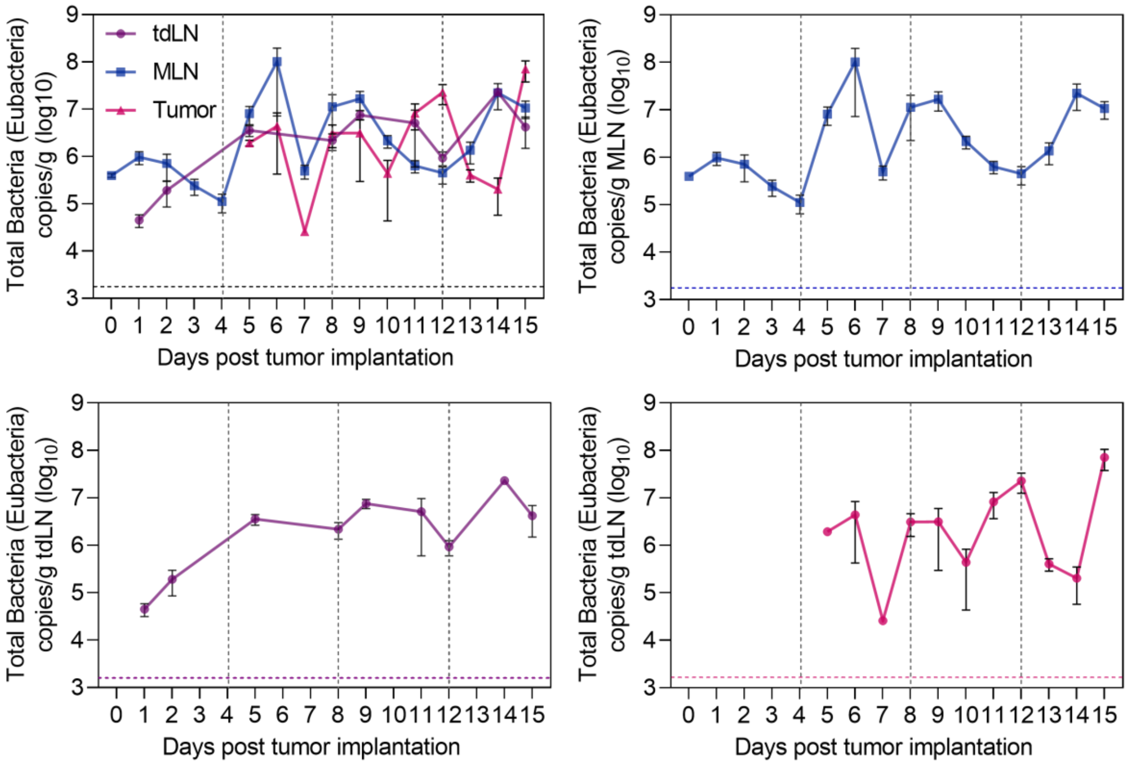

Figure S5. Bacterial levels in MLN, TDLN, and tumor in mice bearing melanoma tumor and receiving ICT

Bacterial abundance (Eubacteria, all bacteria) determined by quantitative-PCR (qPCR)-based quantification of 16s rRNA gene copies of tissue samples recovered from C57BL/6J mice (n=6-8) bearing melanoma tumors and receiving anti-PD-1 and anti-CTLA-4 antibody treatment, as detailed in fig 1D. Dotted line indicates the limit of detection.

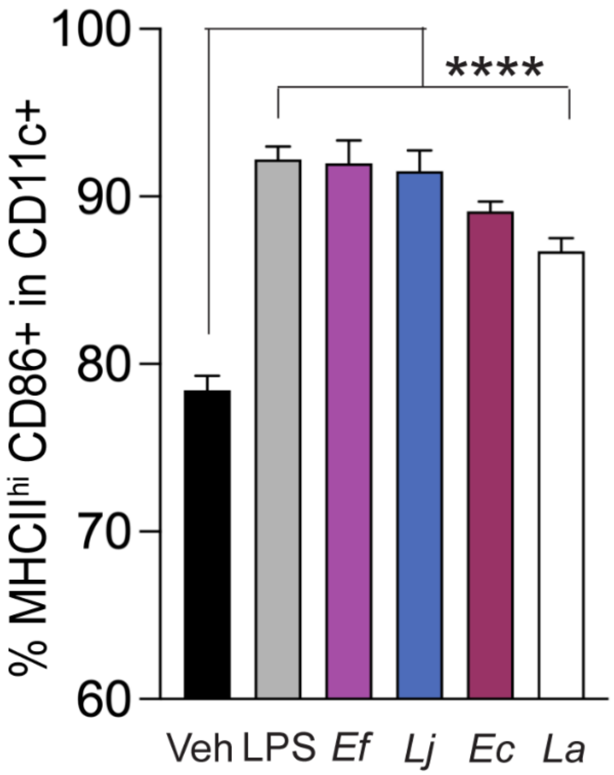

Figure S6. CD86 expression in dendritic cells stimulated with different bacteria.

CD11c+ DCs were isolated from the spleen of C57BL6/J mice (female, 6-8 wks, Jackson) bearing B16-FLT3L tumors. Isolated DCs were stimulated with vehicle (PBS), *E. coli* LPS (Invivogen, 1ug/ml), and the following bacterial lysates (10ug/ml as determined by protein concentration via BCA assay): *Enterococcus faecalis* (*Ef*, clinical isolate from pediatric SCT patient), *Lactobacillus johnsonii* (*Lj*, VPI 7960), *Escherichia coli* (*Ec*, ATCC 10798), and *Lactobacillus acidophilus* (*La*, ATCC 4357) for 6 hours. Surface expression of MHC-II and CD86 were measured by flow cytometry. The proportion of MHC-II high, CD40+ cells among CD11c+ DCs are shown in the plot. Bars represent the mean $\pm$ SEM. Statistical analysis by Mann-Whitney test. ****P<0.0001. All assays were performed in triplicate.

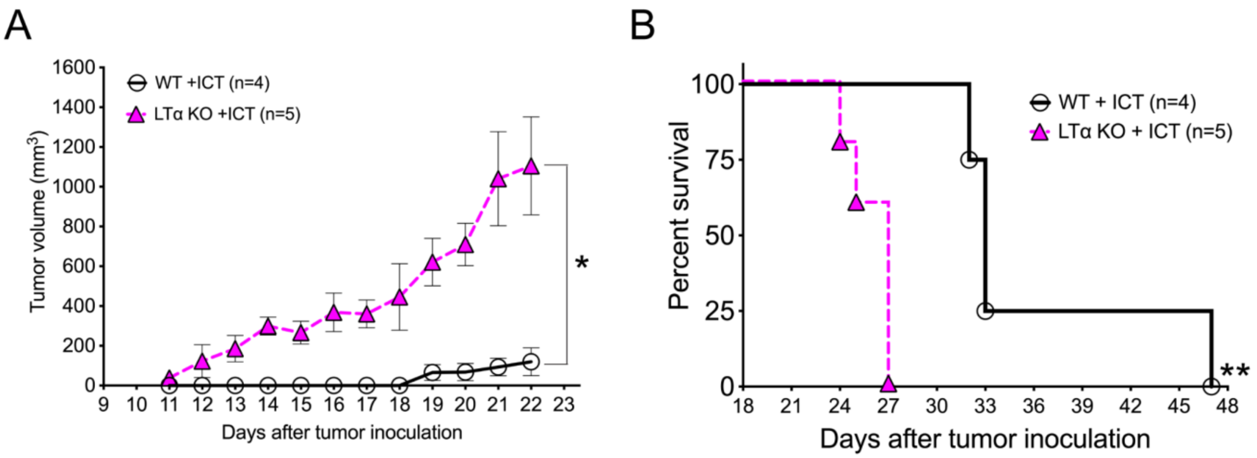

Figure S7. Lymphotoxin alpha knockout (LTα KO) mice have diminished response to ICT compared to co-housed wild-type mice.

(A) Tumor volumes (points equal mean $\pm$ SEM, Mann-Whitney test, *, $P< 0.05$) and (B) survival curves (log-rank test, **, $P<0.01$) of lymphotoxin alpha knockout mice (n=5) and wild-type mice (C57BL/6J, Jackson, female, 6-8, weeks old; n=4) implanted with B16-F10 tumors and treated with ICT (200 ug anti-PD-1 and 200 ug anti-CTLA-4 mAb) on days 4, 8 and 12 after tumor implantation.

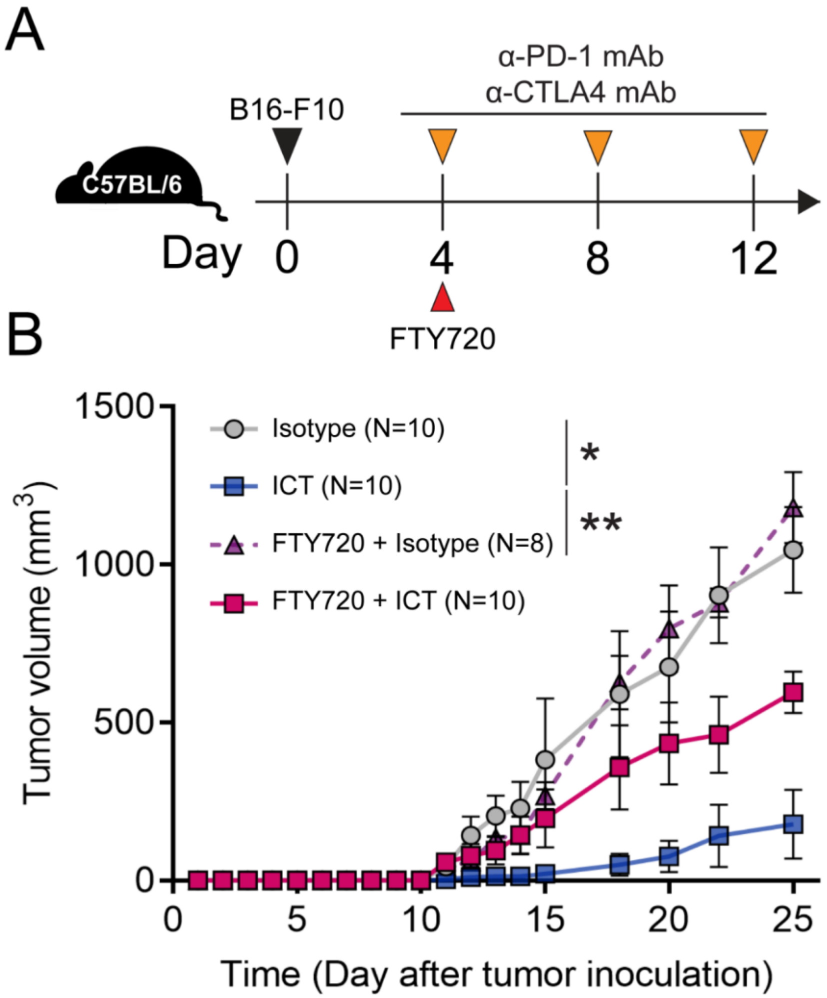

Figure S8. FTY720-induced inhibition of lymphocyte egress from lymph nodes mitigates ICT efficacy.

(A) Schematic overview of protocol. C57BL/6 mice (female, 6-8 wks, Jackson) were subcutaneously implanted with 1 x 10^5 B16-F10 cells. Mice were injected intraperitoneally with ICT (200 µg anti-PD-1 and 200 µg anti-CTLA-4 mAb) on days 4, 8, and 12 post tumor inoculation. A single dose of FTY720 (Fingolimod, 0.3 mg/kg) was injected intraperitoneally on day 4 post tumor inoculation. n=8-10 per group.

(B) Tumor growth of mice treated ± ICT ± FTY720. Points represent the mean ± SEM. Statistical analysis by Mann-Whitney test. *P<0.05, **P<0.01.

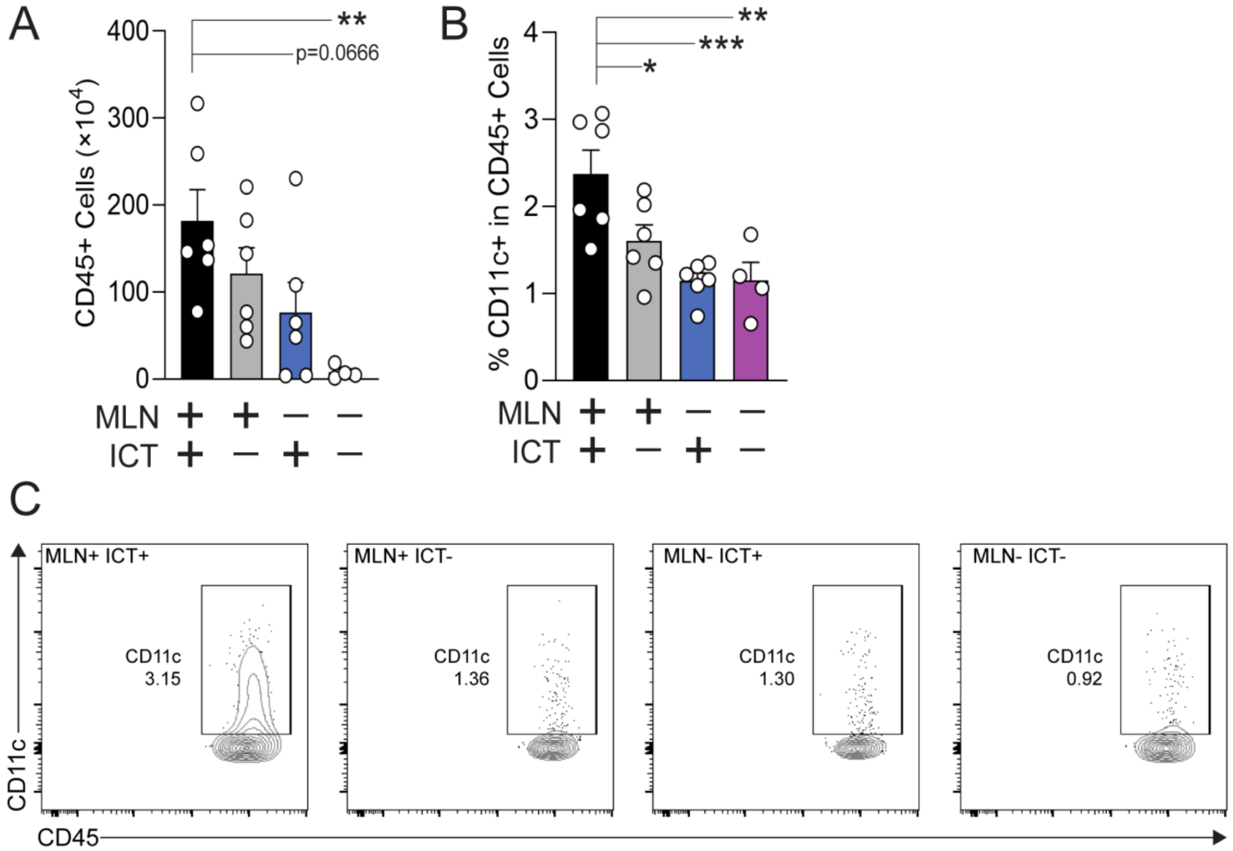

Figure S9. Surgical resection of MLN results in decreased lymphocytes and dendritic cells in TDLN of mice bearing melanoma

Tumor draining lymph nodes (TDLN, right inguinal lymph nodes) were harvested from mice (C57BL/6J, female, 6-8 weeks, n=4-6 per group) bearing B16-F10 tumors ± MLN ± ICT (anti-PD-1 and anti-CTLA-4 mAb) (as in fig. 3F). TDLN cells were counted and proportion of CD45+ and CD11c+ cells were determined by flow cytometry.

(A) Quantification of CD45+ cells in the TDLN.

(B) Proportion of CD11c+ dendritic cells among total CD45+ cells.

(C) Representative flow cytometry plot of CD11c+ dendritic cells among TDLN CD45+ cells.

Points represent values from individual mice. Bars represent the mean ± SEM. Statistical analysis by Mann-Whitney test. *P<0.05, **P<0.01, ***P<0.001.

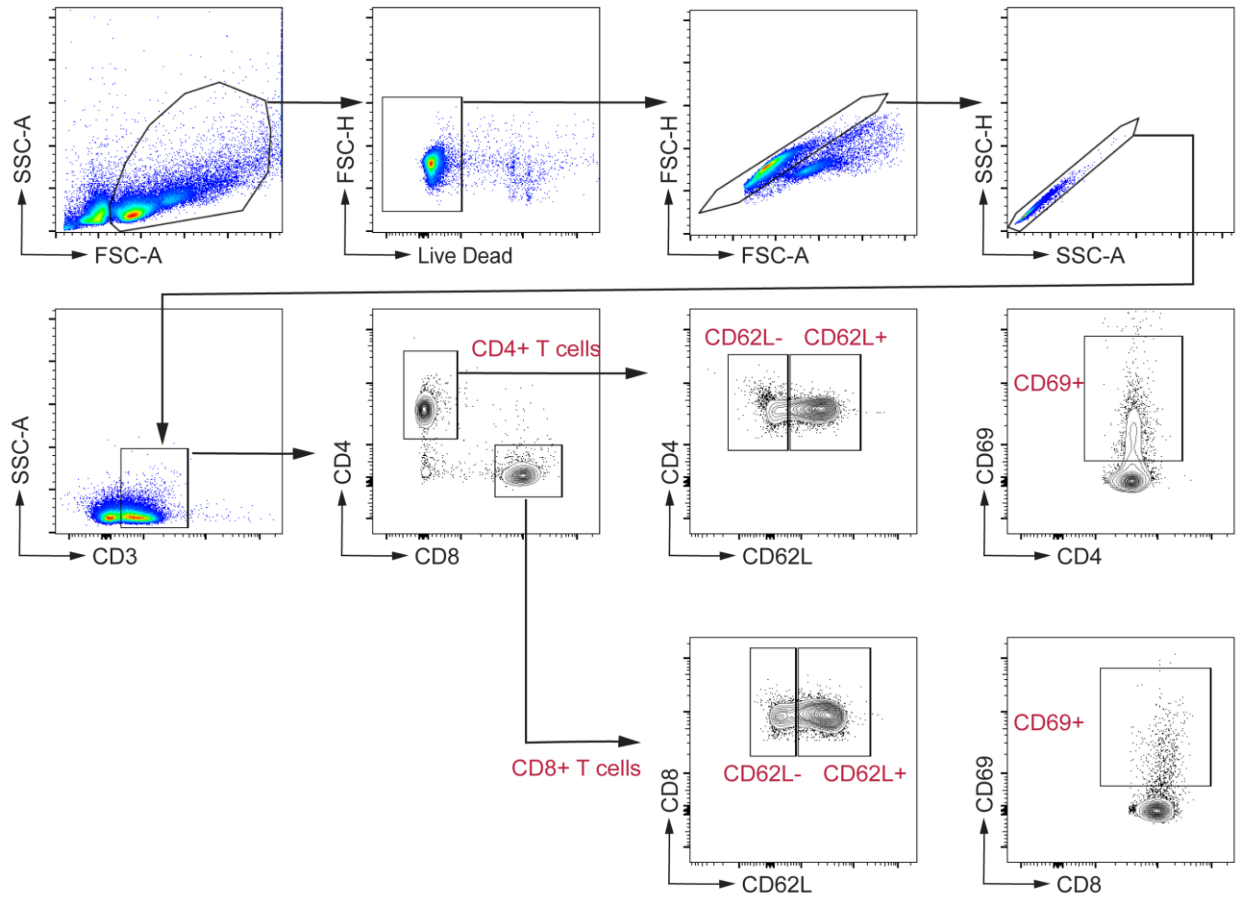

Figure S10. Flow cytometry gating strategy for profiling TDLN T-cell phenotypes.
Cells from the tumor-draining lymph nodes (right inguinal lymph nodes) were isolated and analyzed by flow cytometry. CD62L and CD69 expression within CD4+ T cells (live CD45+CD3+CD4+) and CD8+ T cells (live CD45+CD3+CD8+) were gated as shown. Representative plots are presented here and quantification of CD62L and CD69 expression on each cell population is shown in fig. 3 G-J.

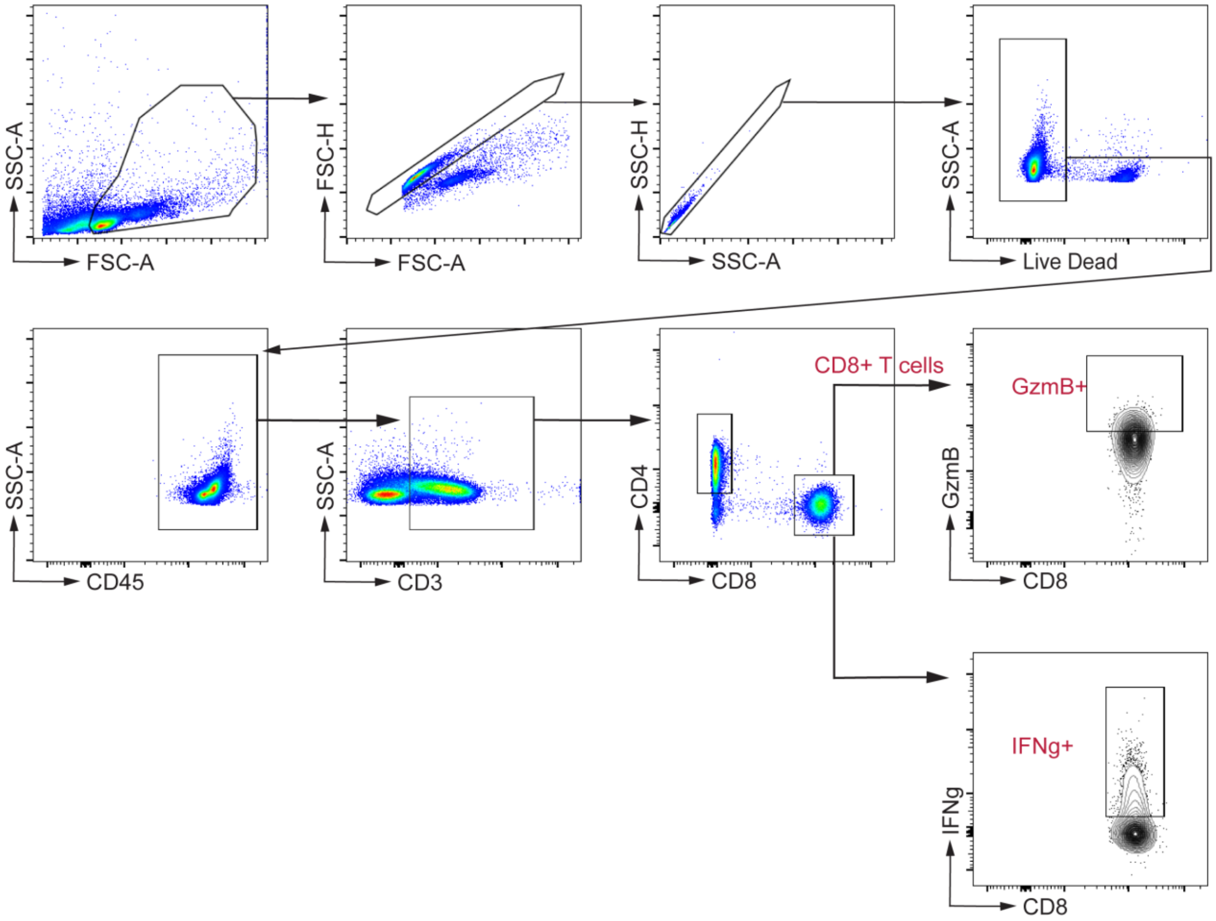

Figure S11. Flow cytometry gating strategy for assessing TDLN T cell cytokine expression. Cells from the tumor-draining lymph nodes were isolated and analyzed by flow cytometry. Intracellular expression of granzyme B (GzmB) and interferon-gamma (IFNg) within CD8+ T cells (live CD45+CD3+CD8+) were gated as shown. Representative plots are presented here and quantification of GzmB and IFN-γ expression on CD8+ T cell population is shown in fig. 3 K and L.

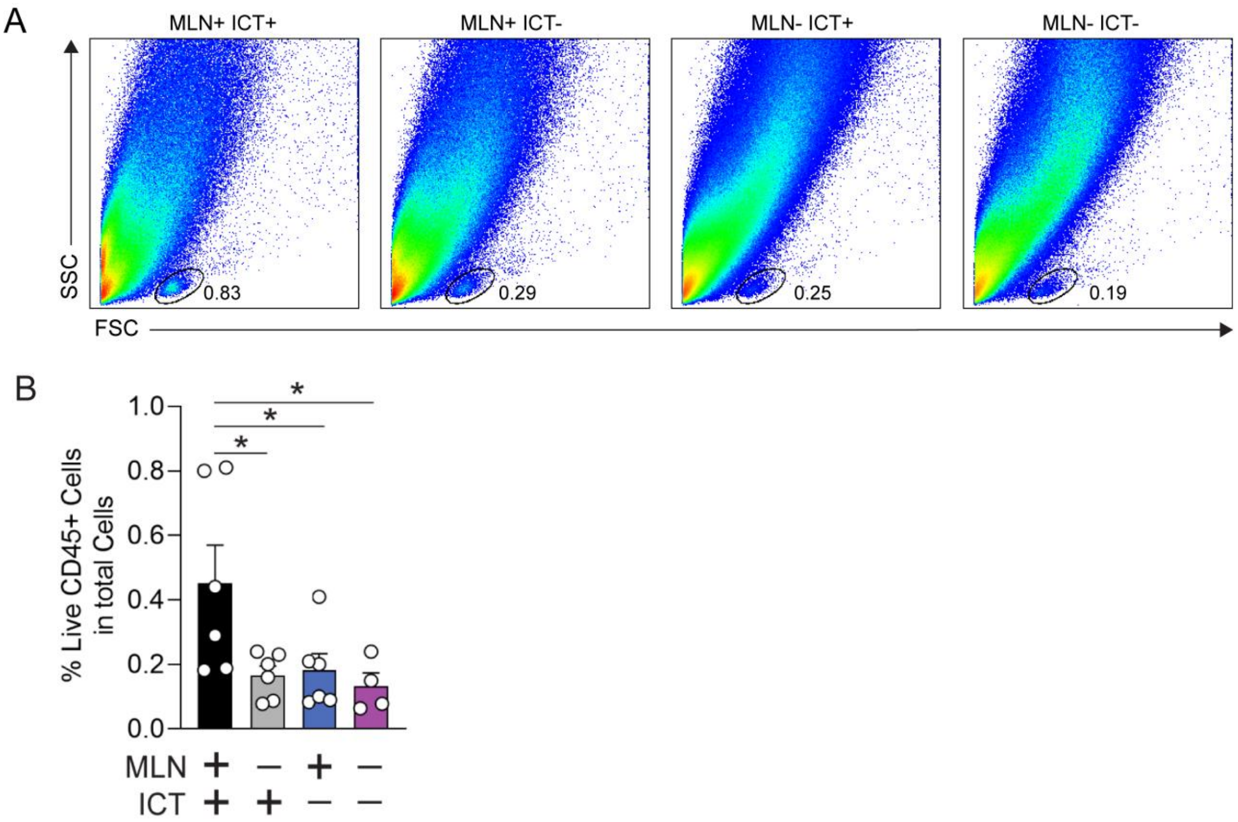

Figure S12. MLN resection results in decreased leukocyte infiltration in tumors of mice bearing melanoma tumors and receiving ICT.

Tumor-infiltrating leukocytes were isolated from B16-F10 tumors from C57BL/6J mice (female, 6-8 weeks) ± MLN (via surgical resection) ± ICT (anti-PD-1 and anti-CTLA-4 mAb) (as in fig. 3F). The proportion of the tumor-infiltrating leukocytes (live CD45+) was analyzed by flow cytometry.

(A) Representative flow plot for lymphocyte gating. More than 98% of the gated cells are live, CD45+ cells (Figure S13.)

(B) Proportion of CD45+ cells among total cells.

Points represent values from individual mice. n=4-6 per group. Bars represent the mean ± SEM. Statistical analysis by Mann-Whitney test. *P<0.05, **P<0.01, ***P<0.001.

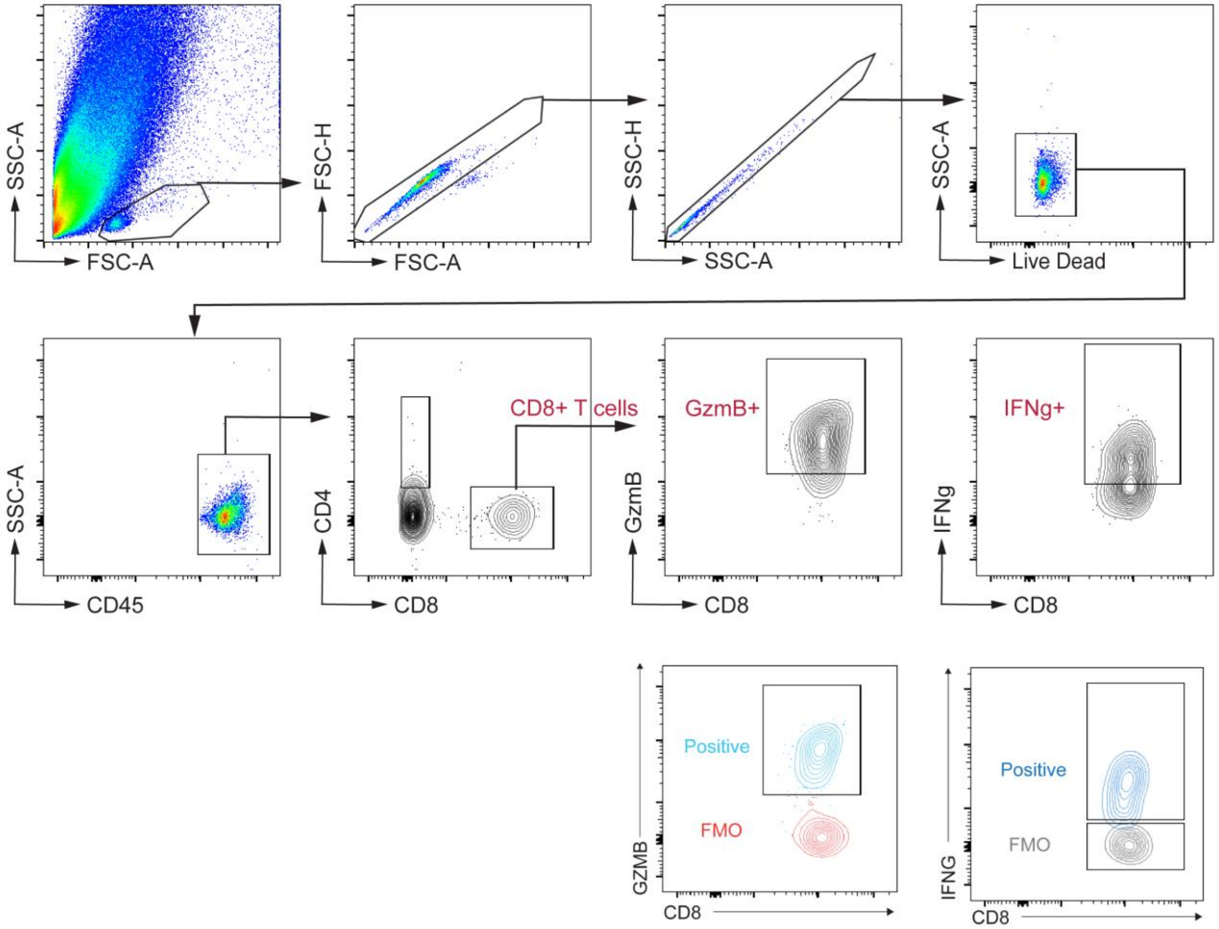

Figure S13. Flow cytometry gating strategy for assessing tumor infiltrating leukocyte cytokine expression.

Tumor-infiltrating leukocytes (CD45+) were isolated and analyzed by flow cytometry. Intracellular expression of granzyme B (GzmB) and interferon-gamma (IFNg) within CD8+ T cells (live CD45+CD8+) were gated as shown. Representative plots are presented here and quantification of GzmB and IFNg expression on CD8+ T cell population is shown in fig. 3M and N.

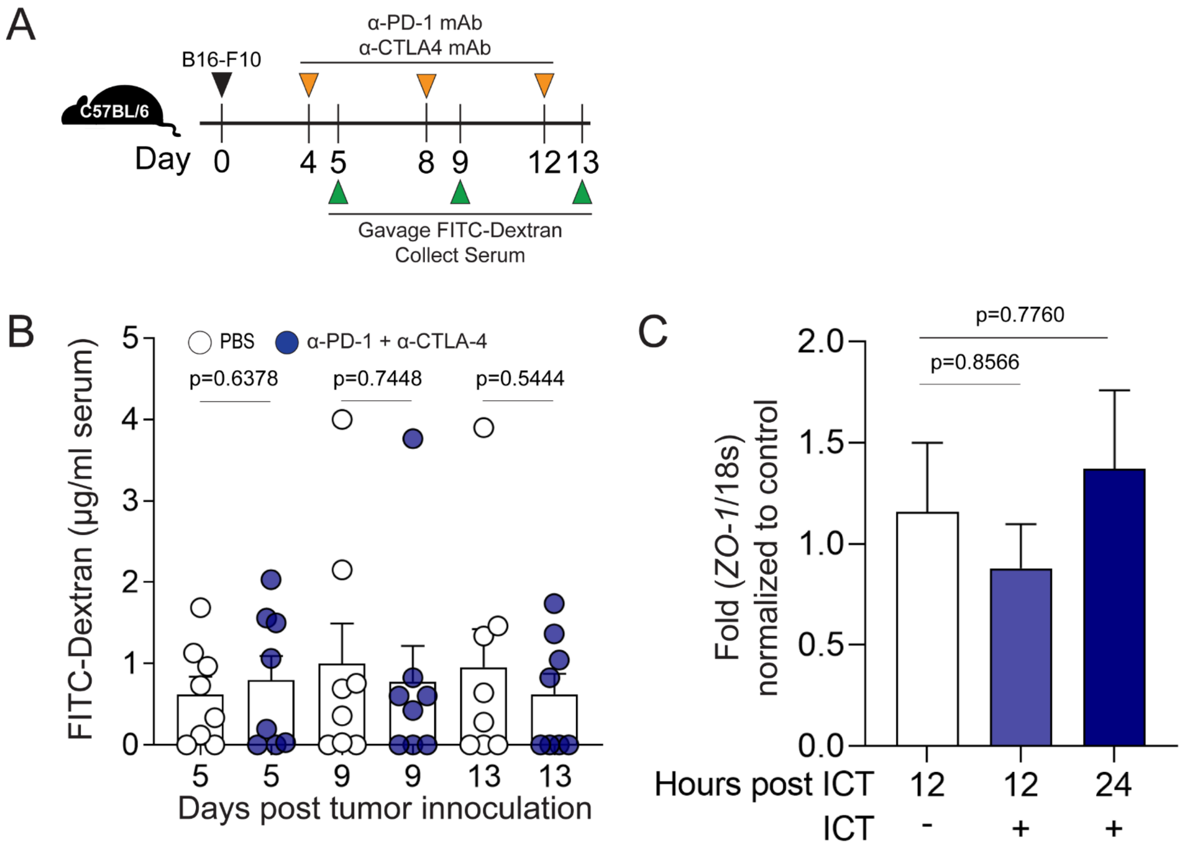

Figure S14. Gastrointestinal barrier function assays in mice bearing melanoma tumors and receiving anti-PD-1 and anti-CTLA-4 antibody therapy.

(A) Schematic overview of protocol. C57BL/6 mice (female, 6-8 wks, Jackson) were subcutaneously implanted with $1x10^5$ B16-F10 cells. Mice were injected intraperitoneally with ICT (200µg anti-PD-1 and 200µg anti-CTLA-4 mAb) on days 4, 8, and 12 post tumor inoculation. Mice were fasted overnight and then orally gavaged with FITC-Dextran (4kD, 500mg/kg, Sigma). Serum was obtained 4 hours after gavage. Serum FITC-Dextran level was measured at an excitation wavelength of 485 nm and an emission wavelength of 528 nm.

(B) Serum FITC-Dextran levels in mice. n=8 per group. Points represent results from individual animals. Bars represent the mean ± SEM. Statistical analysis by unpaired t-test.

(C) Colonic expression of zonulin (*ZO-1*) in mice after receipt of ICT. Colons were collected 12 hours and 24 hours post ICT. mRNA expression of tight junction protein Zonula occludens-1, *ZO-1*, was measured by quantitative-PCR and normalized to 18s rRNA expression level. n=4 per group. Bars represent the mean ± SEM. Statistical analysis by one-way ANOVA.

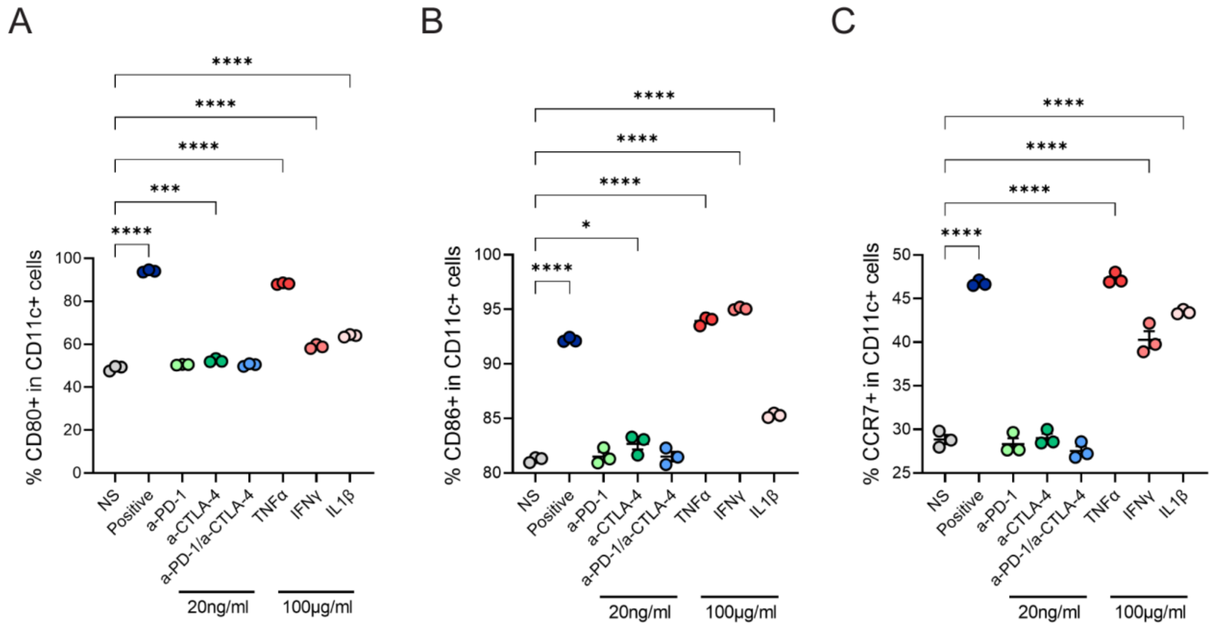

Figure S15. TNF-α, IFN-γ, and/or IL-1-β directly induce murine DC activation, whereas ICT (a-PD1, a-CTLA-4, and a-PD-1/a-CTLA-4) does not

CD11c+ dendritic cells isolated from wild-type C57BL/6 mice (female, 6-8 wks, Jackson) were stimulated with cytokines or immune checkpoint inhibitors, anti-PD-1 and anti-CTLA-4 mAbs, for 18 hours. Surface expression of costimulatory receptors (A) CD80, (B) CD86 and (C) migratory receptor CCR7 were measured by flow cytometry. Statistical analysis by One-way Anova test. *P<0.05, **P<0.01, ***P<0.001, ****P<0.0001. All assays performed in triplicate. NS: Not stimulated.

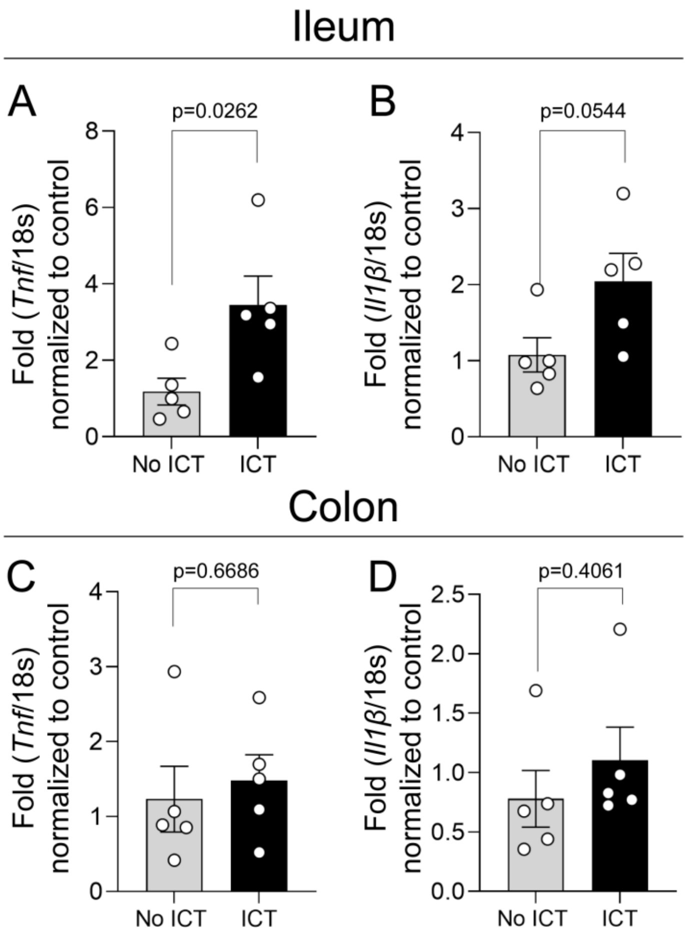

Figure S16. anti-PD-1 and anti-CTLA-4 therapy induces intestinal expression of TNF-α and IL-1β in mice.

Ileum and colonic tissue was collected from C57BL6/J mice (Jackson, female, 6-8 weeks) bearing B16-F10 melanoma tumors and treated + ICT (200 ug anti-PD-1 and anti-CTLA-4 mAb). Tissue samples were harvested one day after the first ICT dose (D+4 after tumor implantation).

(A) *Tnf* and (B) *Il1b* mRNA expression in ileums from mice treated + ICT

(C) *Tnf* and (D) *Il1b* mRNA expression in colons from mice treated + ICT

n=5 per group. Points represent values from individual animals. Bars represent the mean $\pm$ SEM. Statistical analysis by t-test. *P<0.05.

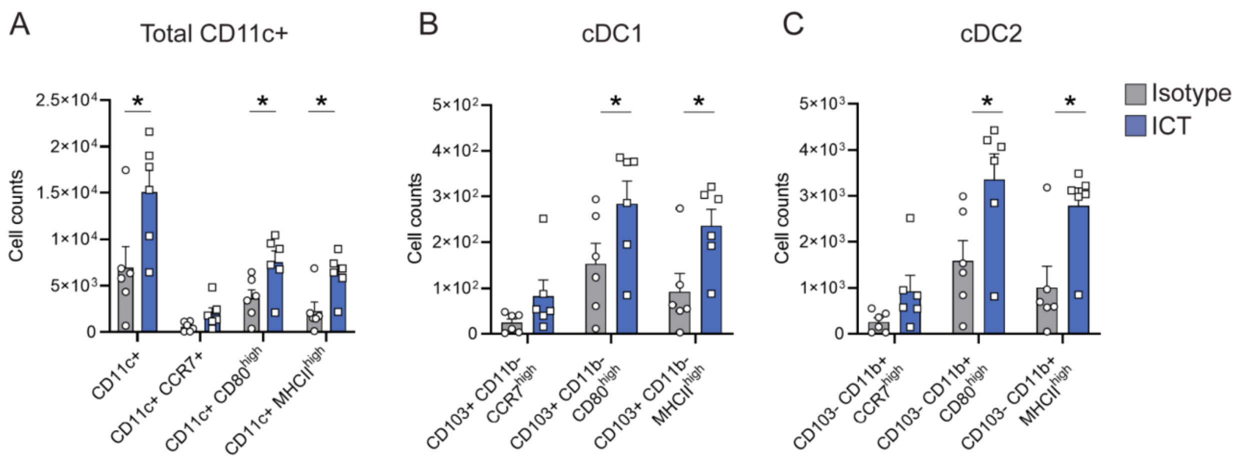

Figure S17. ICT induces lamina propria dendritic cell activation (CD80, MHC II) and migratory potential (CCR7)

C57BL/6 mice (female, 6-8 wks, Jackson) were inoculated with 1×10^5 B16-F10 cells subcutaneously in the right flank. 3 doses of 200µg anti-PD-1 and 200µg anti-CTLA-4 mAb (ICT) or isotype antibodies were injected intraperitoneally (n=6 per group). Lamina propria (LP) leukocytes were isolated from whole small intestine. Expression of DC activation markers CD80 and MHC II and migratory potential marker CCR7 as determined by flow cytometry in total CD11c+ DCs

conventional DC subset 1 (cDC1; CD103+CD11b-)

conventional DC subset 2 (cDC2; CD103-CD11b+)

Points represent values from individual animals. n=6 per group. Bars represent the mean ± SEM. Statistical analysis by unpaired t-test. *P<0.05.

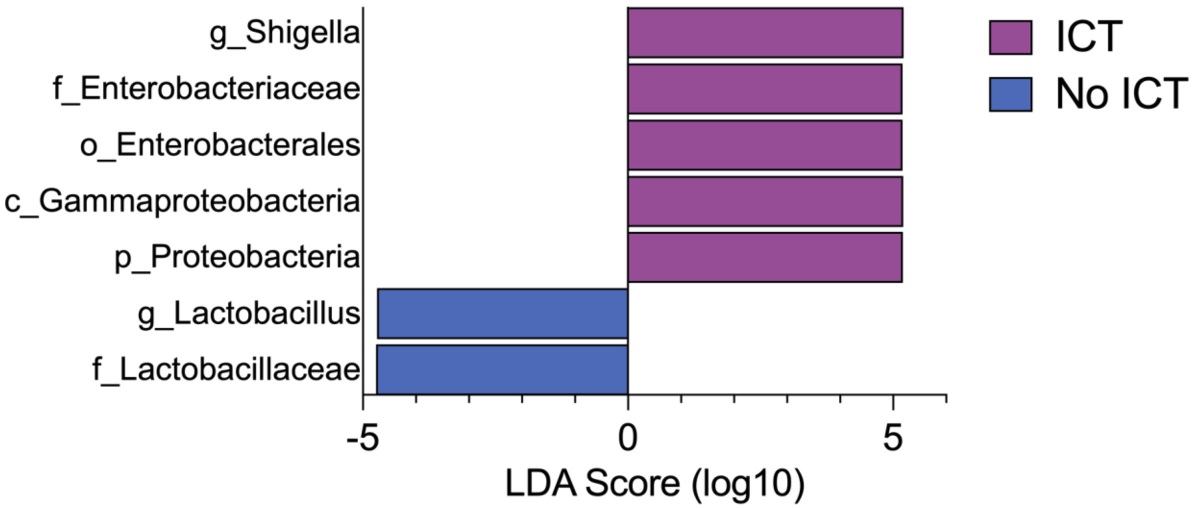

Figure S18. Comparison of dendritic cell microbiomes from mice bearing melanoma tumors and treated with or without anti-PD-1 and anti-CTLA-4 antibody treatment.
Microbiome composition determined by analysis of 16S rRNA sequencing (V4 region) of dendritic cells recovered from MLNs in C57BL/6J mice bearing melanoma tumors treated with or without anti-PD-1 and anti-CTLA-4 antibody treatment (ICT). Differential bacterial taxonomic abundance between groups was analyzed by linear discriminant analysis effect size (LEfSe) projected as histograms. All listed bacterial groups (phylum (p), class (c), order (o), family (f), or genus (g)) were significantly enriched (>2 log-fold increase in linear discriminate analysis, LDA, score and $P<0.05$, Kruskal-Wallis test).

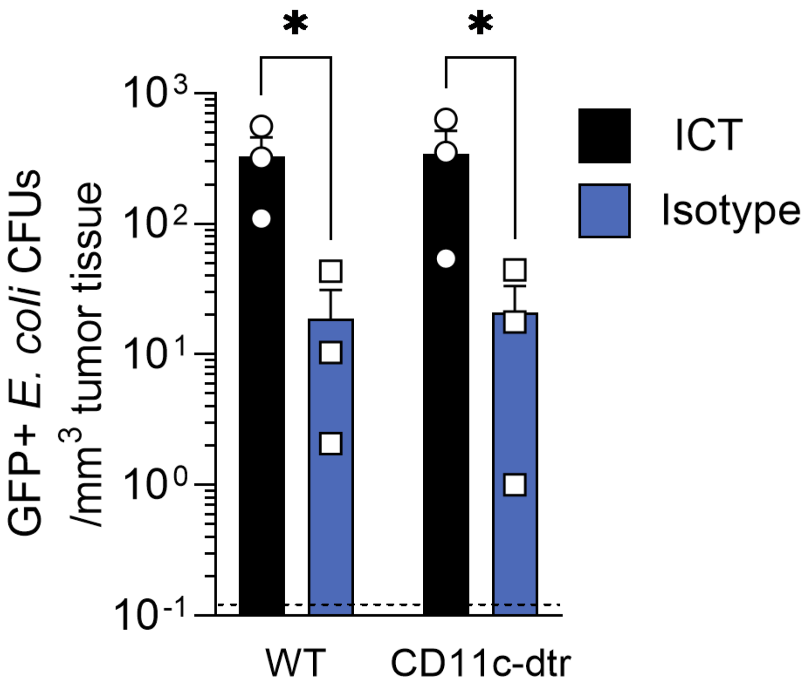

Figure S19. Bacterial levels in tumors of wildtype and CD11c-dtr mice after intra-MLN bacterial injection.
CD11c-dtr mice (female, 6-8 wks, Jackson) were injected with 100 ng diphtheria toxin (DT) intraperitoneally on day 3 post tumor implantation to deplete CD11c+ DCs. Wild-type C57BL/6 and DT-treated CD11c-dtr mice were implanted with B16-F10 tumor. Mice with comparable tumor volumes were randomized before the ICT. 3 doses of ICT or isotype controls were injected intraperitoneally. 1×10^7 GFP+ *E. coli* was injected directly into the MLN. Tumor tissue was collected 24 hours post *E. coli* injection. Tumor homogenates were spread on TSA-Kanamycin Agar plates. GFP+ colonies were enumerated after 24 hours of incubation at 37°C. n=3 per group. Squares represent values from individual mice. Bars represent the mean $\pm$ SEM. Statistical analysis by t-test. $^*P<0.05$.

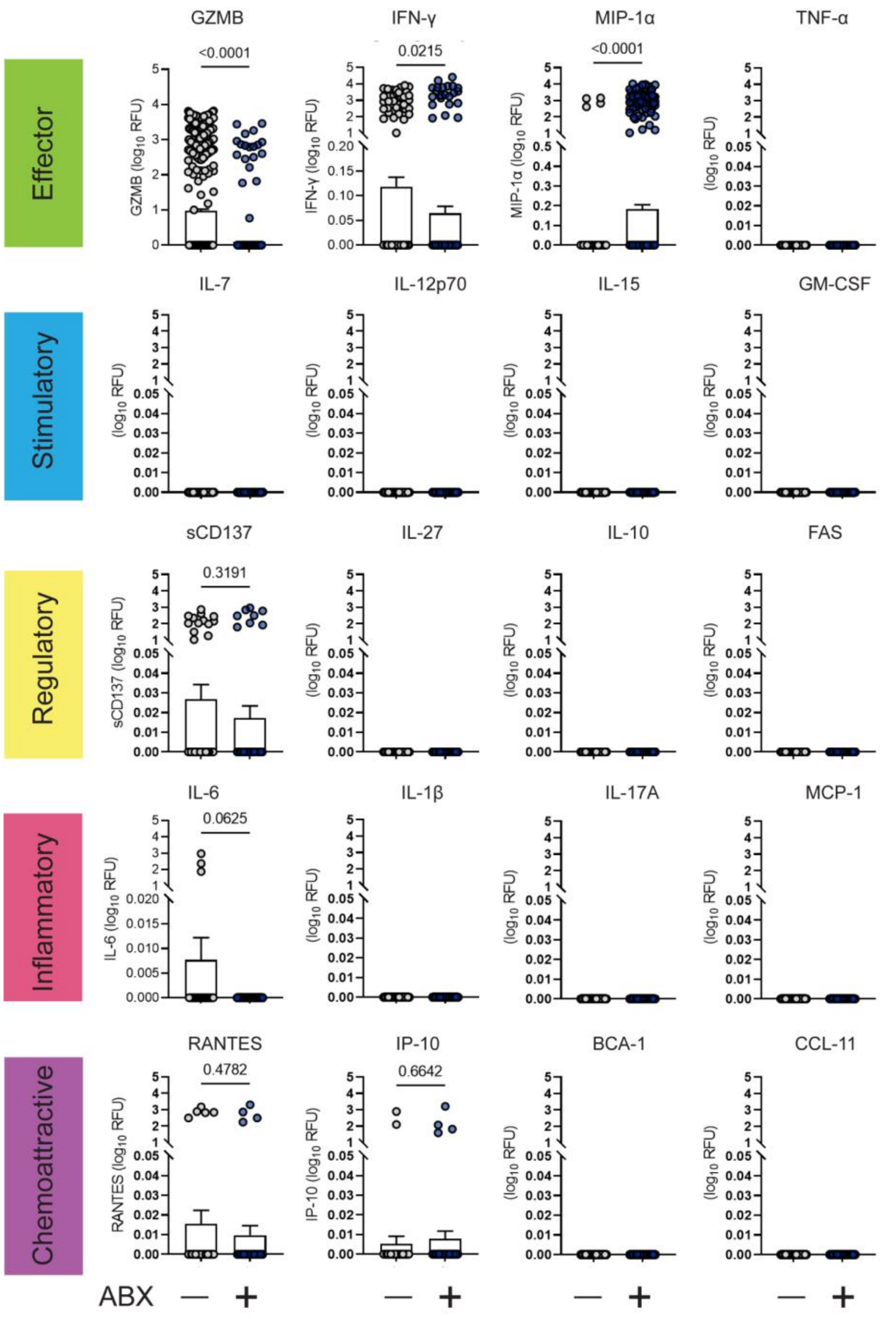

Figure S20. Secreted cytokine profiling of MLN CD8+ T cells isolated from mice ± antibiotics C57BL/6 mice (female, 6-8 wks, Jackson) were treated + antibiotics (ABX, 2 mg/ml streptomycin and 1500 U/ml penicillin G in drinking water) for 7d before B16-F10 tumor inoculation. Mice were treated with 200μg anti-PD-1 and 200μg anti-CTLA-4 mAb intraperitoneally on days 4, 8, and 12 after tumor implantation. CD8+ T cells from 3 mice were pooled per group. Secretory cytokine profiles of CD8+ T-cells isolated from MLN of mice ± ABX ± ICT, as determined by single-cell multiplex cytokine profiling (Isoplexis IsoSpark; 28-plex mouse adaptive immune IsoCode chip panel). Points represent values from individual CD8+ T cells. Bars represent the mean ± SEM. Statistical analysis by t-test.

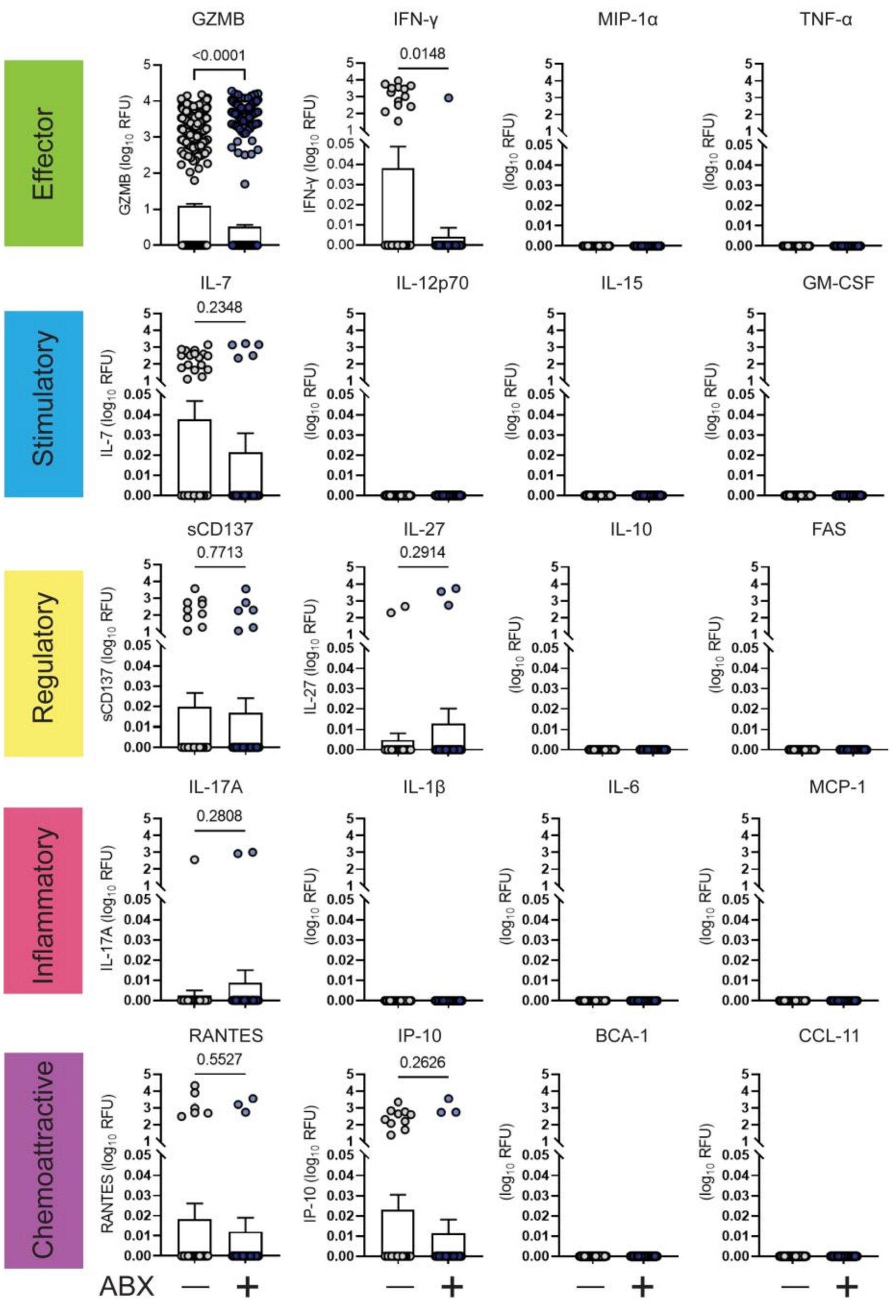

Figure S21. Secreted cytokine profiling of TDLN CD8+ T cells isolated from mice ± antibiotics

C57BL/6 mice (female, 6-8 wks, Jackson) were treated ± antibiotics (ABX, 2 mg/ml streptomycin and 1500 U/ml penicillin G in drinking water) for 7d before B16-F10 tumor inoculation. Mice were treated with 200µg anti-PD-1 and 200µg anti-CTLA-4 mAb intraperitoneally on days 4, 8, and 12 after tumor implantation. CD8+ T cells from 10 mice were pooled per group. Secretory cytokine profiles of CD8+ T-cells isolated from TDLN of mice ± ABX ± ICT, as determined by single-cell multiplex cytokine profiling (Isoplexis IsoSpark; 28-plex mouse adaptive immune IsoCode chip panel). Points represent values from individual CD8+ T cells. Bars represent the mean ± SEM. Statistical analysis by t-test.

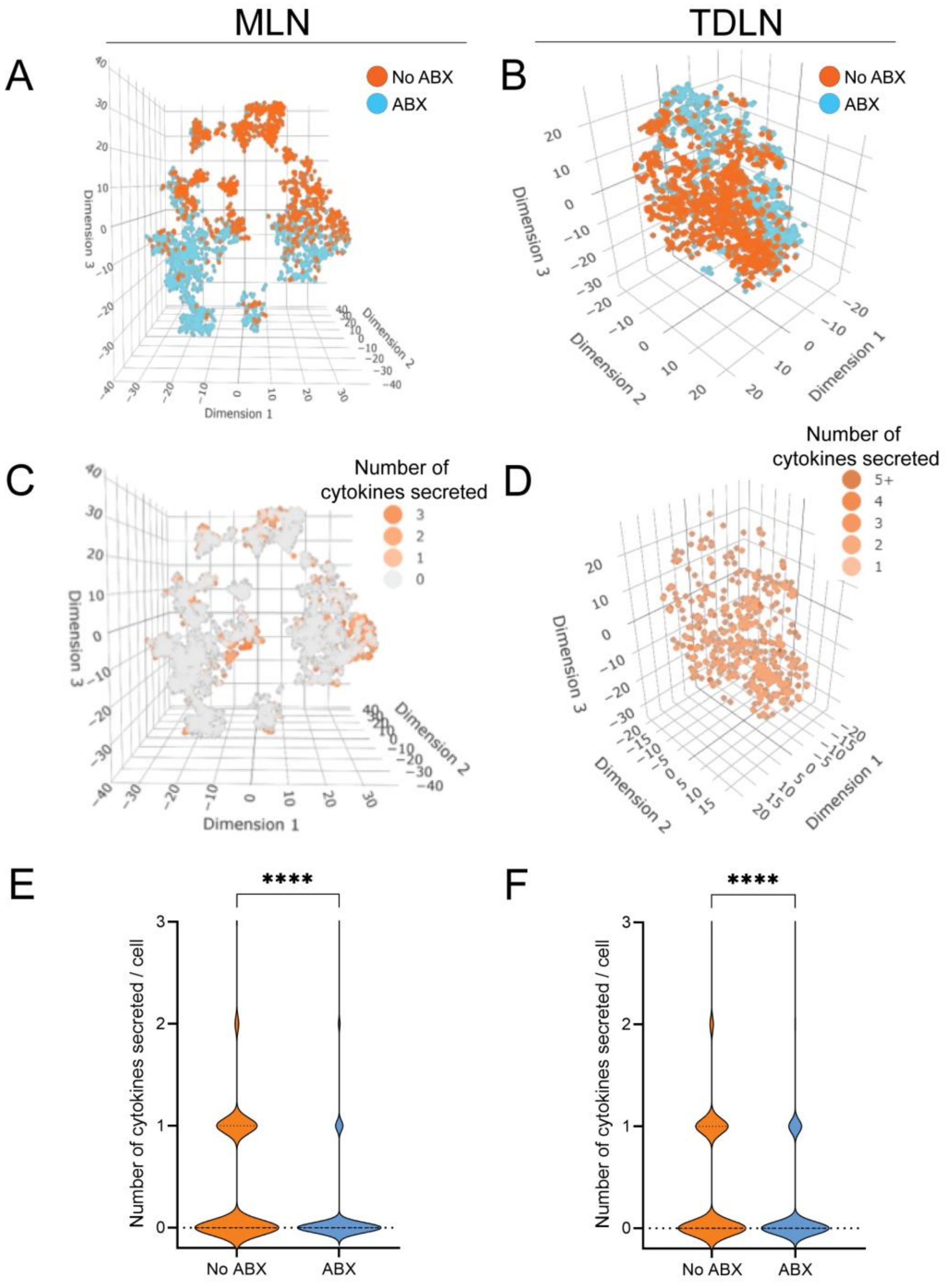

Figure S22. Cytokine secretion profile of CD8+ T cells isolated from MLN and TDLN of mice treated with ICT and with or without antibiotics.

Three-dimensional t-distributed stochastic neighbor embedding (t-SNE) plot of secretory cytokine profiles of CD8+ T-cells isolated from (A) MLN and (B) TDLN of B16-F10 melanoma tumor bearing mice (C57BL/6J, Jackson, female, 6-8 wks, n=3 for MLN group, n=10 for TDLN group) treated with ICT + antibiotics (ABX, 2 mg/ml streptomycin and 1500 u/ml penicillin G). Representation of polyfunctional CD8 T cells from (C) MLN and (D) TDLN in the 3D-tSNE plot. The number of cytokines secreted per cell among (E) MLN and (F) TDLN CD8+ T cells. Statistical analysis by Mann-Whitney test. ****P<0.0001.

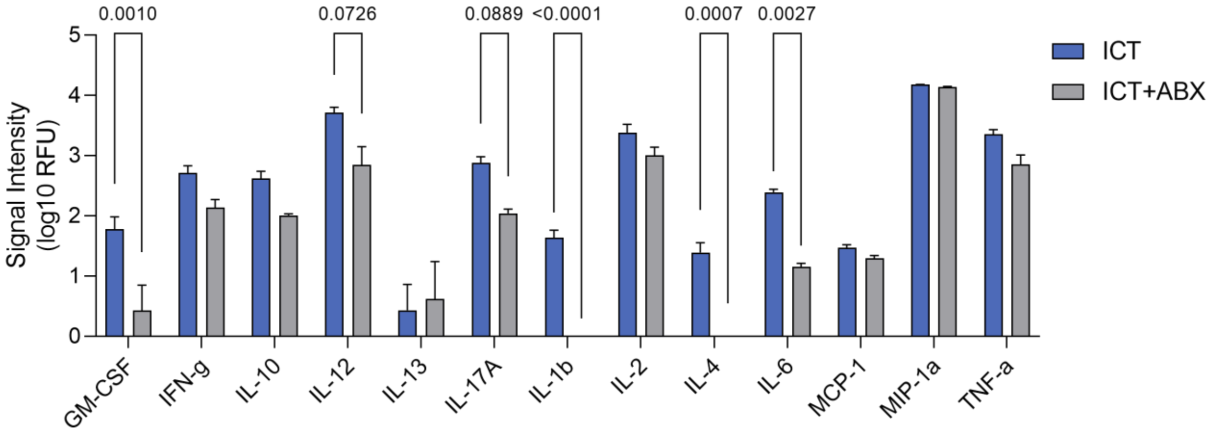

Figure S23. Cytokine secretion profile of TDLN dendritic cells of mice treated with ICT and with or without antibiotics.

C57BL/6 mice (female, 6-8 wks, Jackson) were treated ± antibiotics (ABX, 2 mg/ml streptomycin and 1500 U/ml penicillin G in drinking water) for 7d before B16-F10 tumor inoculation. Mice were treated with 200µg anti-PD-1 and 200µg anti-CTLA-4 mAb intraperitoneally on days 4, 8, and 12 after tumor implantation. n=10 mice per group. Mice were sacrificed on day 13 and CD11c+ dendritic cells isolated from 5 mice were pulled into one sample for the downstream cytokine secretion profiling (two pooled samples per group). Isolated CD11c+ dendritic cells were then stimulated with PMA and Ionomycin for 6 hours and supernatants were collected for multiplex cytokine profiling (Isoplexis IsoSpark; 16-plex mouse inflammation Codeplex chip panel). Bars represent the mean ± SEM. Statistical analysis by one-way ANOVA with Fisher's Least Significant Difference (LSD) test.

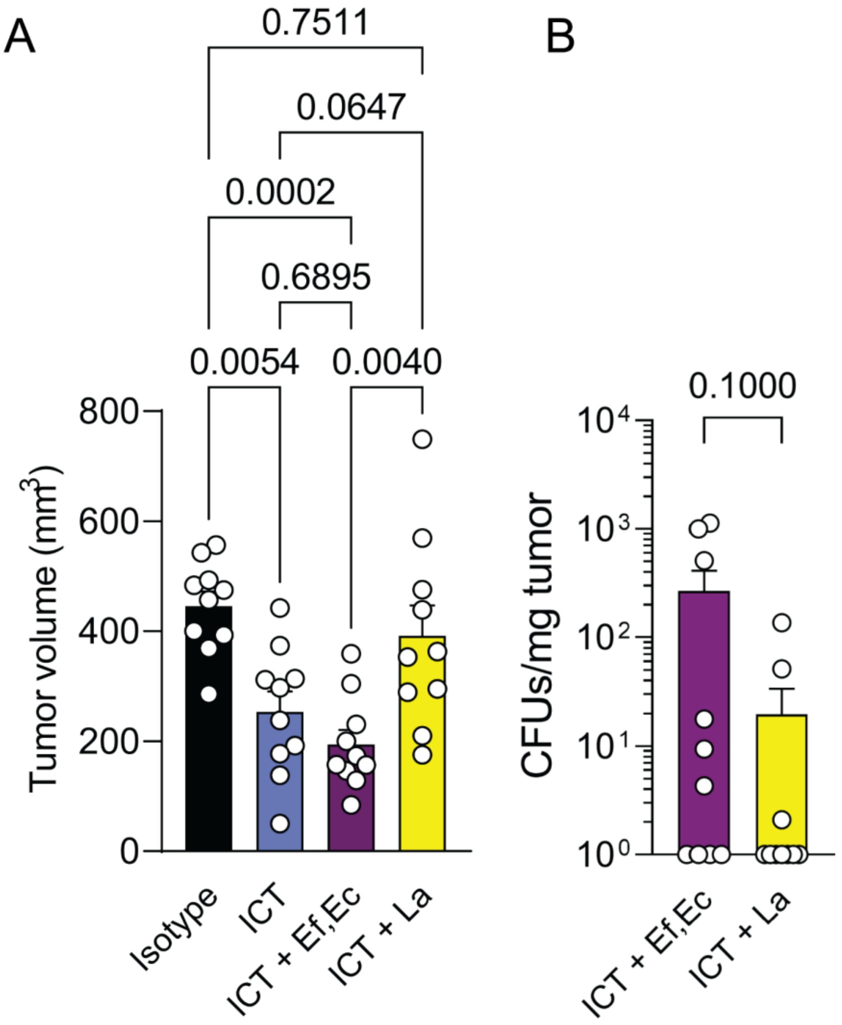

Figure S24. Oral administration of live *Enterococcus faecalis* and *Escherichia coli* induces melanoma tumor volume reduction in mice treated with ICT

C57BL/6 mice (female, 6-8 wks, Jackson) were treated with antibiotics (ABX, 2 mg/ml streptomycin and 1500 U/ml penicillin G in drinking water) for 7 days. Mice were then inoculated with 1×10^5 B16-F10 cells subcutaneously in the right flank and orally gavaged with a total of 2 x 10^8 CFU live bacteria every two days: $1x10^8$ CFU of *E. faecalis* and $1x10^8$ CFU *E.coli*; and 2 x 10^8 CFU of *L. acidophilus*. Mice were treated with 200µg anti-PD-1 and 200µg anti-CTLA-4 mAb intraperitoneally on days 4, 8, and 12 after tumor implantation.

(A) melanoma tumor volume on day 13 post tumor implantation

(B) melanoma tumor bacterial load as determined by enumeration of cultured bacteria from tumor homogenates plated on BHI/Blood and grown aerobically at 37°C for 48 hours

Points represent values from individual mice. n=10 per group. Bars represent the mean + SEM. Statistical analysis by Mann-Whitney test. Ef, *Enterococcus faecalis*. Ec, *Escherichia coli*. La, *Lactobacillus acidophilus*.

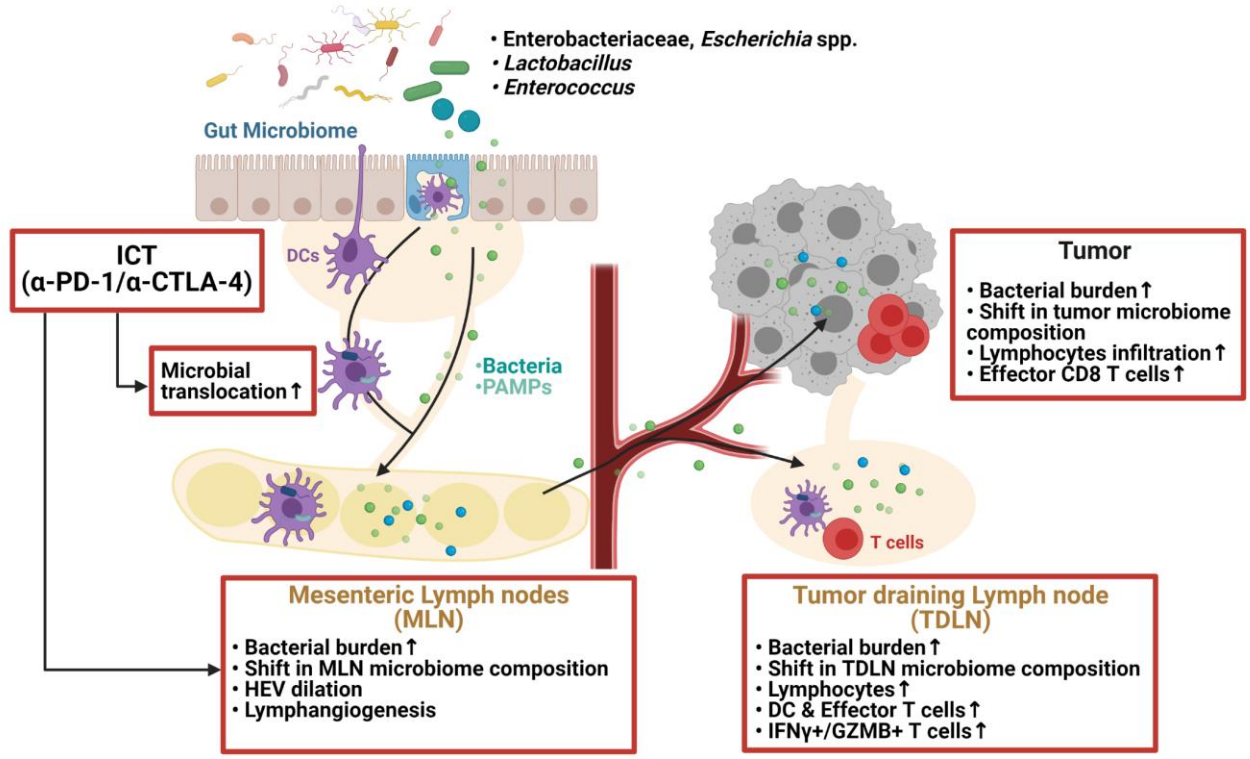

Figure S25. Schema for proposed mechanism

ICT induces 1) DC recruitment into MLN; 2) DC dependent microbial translocation into MLN and 3) MLN remodeling (high endothelial venules, HEV. dilation and subcapsular sinus, SCS, lymphangiogenesis). Bacterial disseminate from MLN to extraintestinal sites, including the tumor and TDLN, possibly via hematogenous spread. Bacteria and bacterial components within the tumor and TDLN augment anti-tumor immunity and ICT efficacy. ICT, immune checkpoint inhibitor therapy. DC; dendritic cells. MLN; mesenteric lymph node. TDLN, tumor draining lymph node. PAMPs, pathogen-associated molecular patterns.

Table S1. Antibodies used for flow cytometry

Table S1. Antibodies used for flow cytometry

Specificity	Clone	Isotype	Fluorochrome	Supplier	Catalog number	Dilution
Live/Dead			Zombie yellow	Biolegend	423104	1/600
CD45	30-F11	Rat IgG2b, κ	Brilliant violet 650	Biolegend	103151	1/400
CD45	30-F11	Rat IgG2b, κ	PE-Cyanine7	Biolegend	103114	1/400
CD3e	145-2C11	Armenian Hamster IgG	APC	Biolegend	100312	1/200
CD4	RM4-5	Rat IgG2a, κ	AlexaFluor700	Biolegend	100536	1/400
CD8a	53-6.7	Rat IgG2a, κ	FITC	Biolegend	100706	1/400
IFN-γ	XMG1.2	Rat IgG1, κ	Pacific Blue	Biolegend	505818	1/200
Granzyme B	QA16A02	Mouse IgG1, κ	PE-Cyanine7	Biolegend	372214	1/200
CD62L	MEL-14	Rat IgG2a, κ	Brilliant violet 510	Biolegend	104441	1/200
CD69	H1.2F3	Armenian Hamster IgG	PE	Biolegend	104508	1/200
CD69	H1.2F3	Armenian Hamster IgG	Brilliant violet 650	Biolegend	104541	1/200
CD11c	N418	Armenian Hamster IgG	FITC	Biolegend	117306	1/200
MHC-II (I-A/I-I	M5/114.15.2	Rat IgG2b, κ	AlexaFluor700	Biolegend	107622	1/400
CD80	16-10A1	Armenian Hamster IgG	Brilliant violet 711	Biolegend	104743	1/200
CD11b	M1/70	Rat IgG2b, κ	PE	Biolegend	101208	1/200
CCR7	4B12	Rat IgG2a, κ	APC	Biolegend	120108	1/200
CD103	2E7	Armenian Hamster IgG	Pacific Blue	Biolegend	121418	1/200
CD86	GL-1	Rat IgG2a, κ	PerCP	Biolegend	105026	1/200
CD40	3/23	Rat IgG2a, κ	APC	Biolegend	124612	1/200

Table S2. Key resources table

Table S2. Key resources table		
**REAGENT or RESOURCE**	**SOURCE**	**IDENTIFIER**
**Antibodies (Other than flow cytometry)**		
Anti-PD-1 (RMP-1, CD270)	BioXcell	Cat# BP0146
Anti-CTLA-4 (9D9, CD152)	BioXcell	Cat# BP0164
Anti-CD3e	Invitrogen	Cat# 16-0031-86
Anti-CD28	Invitrogen	Cat# 16-0281-85
Rat anti-MECA-79	Santa Cruz Biotechnology	SC-19602
**Chemicals, peptides, and recombinant proteins**		
FOXP3/Transcription Factor Staining Buffer kit	Invitrogen	Cat# 00-5521-00
AccuPrime™ Pfx SuperMix	Invitrogen	Cat# 12344040
SsoAdvanced Universal SYBR Green Supermix	Bio-Rad	Cat# 1725274
Caprofen	Animal resource center, UTSW	DMC10-201013-01
Recombinant murine IL-2	Peprotech	Cat# 212-12
Diphtheria Toxin from Corynebacterium diphtheriae	Sigma	Cat# D0564
Ultrapure LPS, E. coli 0111:B4	Invitrogen	Cat# tlrl-3pelps
**Experimental models: Organisms/strains**		
Enterococcus faecalis	This study	
Lactobacillus johnsonii VPI 7960	ATCC	ATCC 33200
Lactobacillus acidophilus ATCC 4357	ATCC	ATCC 4357
Escherichia coli ATCC 10798	ATCC	ATCC 10798
Escherichia coli pZe21-RBSmod + sfGFP	Dr. Kevin Forsberg	
B16-F10	ATCC	ATCC CRL-6475; RRID:CVCL_0159
B16-FLT3L	Dr. Chandrashekhar Pasare	RRID:CVCL_IJ12
**Critical commercial assays**		
Pierece BCA assay kit	Thermo Scientific	REF 23225
CD11c microbeads ultrapure, mouse	Miltenyi Biotec	Cat# 130-125-835
**Software and algorithms**		
FlowJo (v.10.8.0)	BD Biosciences	https://www.flowjo.com/
QIIME 2	Bolyen et al., 2019	https://qiime2.org
GraphPad Prism 9	GraphPad Software	https://www.graphpad.com
ImageScope (v. 12.3)	Leica Biosystems	https://www.leicabiosystems.com/
OncoLnc	J Anaya, 2016	http://www.oncolnc.org/
**Other**		
Screw-cap microfuge tube (2ml)	Fisher Scientific	REF 72.693.005
Borosilicate glass beads (5mm)	Sigma	Cat# Z143944
Pack-Rectangular Jar (2.5L)	Mitsubishi Gas Chemical	Order# 50-25
Anaerobic gas pack	Mitsubishi Gas Chemical	Order# 10-01
Absorbable sutures (5/0 PGA)	Covetrus	Cat# 031995
Nonabsorbable sutures (5/0 Monofilament nlylon)	Covetrus	Cat# 056919
Vetrinary surgical adhesive	Covetrus	Cat# 031477
Triple antibiotic ointment (Bacitracin zinc, neomycin sulfate, and polymyxin b sulfate ointment	Taro Pharmaceutical	NDC 51672-2120-2
Sterile cell strainer (70μm)	Fisher Scientific	Cat# 22363548
Syringe (10ml)	BD	REF 302995
Petridish	Falcon	Cat# 351029
gentleMACS™ Dissociator	Miltenyi Biotec	Cat# 130-093-235
Tumor dissociation kit, mouse	Miltenyi Biotec	Cat# 130-096-730
LS column	Miltenyi Biotec	Cat# 130-042-401
BD Vacutainer SST tube	BD	REF 367983
